# From Structure to Performance: The Critical Role of DNTT Morphology in Organic TFTs

**DOI:** 10.1021/acsami.5c07824

**Published:** 2025-06-18

**Authors:** Mattia Scagliotti, Antonio Valletta, Silvia Milita, Luigi Mariucci, Gino Giusi, Hussam Bouaamlat, Ari Paavo Seitsonen, Paolo Branchini, Luca Tortora, Matteo Rapisarda

**Affiliations:** † 9327 CNR-IMM, Institute for Microelectronics and Microsystems IMM, Rome 00133, Italy; ‡ 228704 CNR-ISMN, Institute of Nanostructured Materials ISMN, Bologna 40129, Italy; § 119625 University of Messina, Engineering Department, Messina 98158, Italy; ∥ Roma Tre University, Department of Science, Rome 00146, Italy; ⊥ INFN Roma Tre, Rome 00146, Italy; # Department of Chemistry, 26909 École Normale Supérieure, Paris 75005, France

**Keywords:** organic thin-film transistor, semiconductor/dielectric interface, atomic force microscopy (AFM), X-ray diffraction (XRD), density functional theory (DFT), low-frequency noise measurements, TCAD simulations, organic phototransistor (OPT)

## Abstract

The electrical performance of organic thin-film transistors (OTFTs) based on DNTT as the semiconductor active layer (DNTT, which stands for dinaphtho [2,3-b:2′,3′-*f*] thieno [3,2-*b*] thiophene) is investigated and related to the structural properties of the organic films grown on SiO_2_ and Cytop substrates. Conventional current–voltage measurements and high-sensitivity low-frequency measurements show a lower mobility and correspondingly higher defect density for DNTT/SiO_2_ devices. Morphological and structural characterizations of DNTT films grown on the two dielectrics were performed using atomic force microscopy (AFM) and X-ray diffraction (XRD), revealing a highly ordered crystalline structure. Consistent with DFT simulation results, morphological analysis shows that the semiconductor films are layered, with DNTT molecules arranged with their longest axis perpendicular to the substrate. However, in only DNTT/SiO_2_ films, some molecules were found to be ordered and arranged parallel to the substrate. This “horizontal” orientation causes differences in charge transport properties in the semiconductor films grown on SiO_2_, reducing the field-effect mobility. TCAD simulations indicate that this horizontal molecular orientation can be modeled as highly defective regions at semiconductor grain boundaries, consistent with low-frequency noise measurement results.

## Graphical abstract

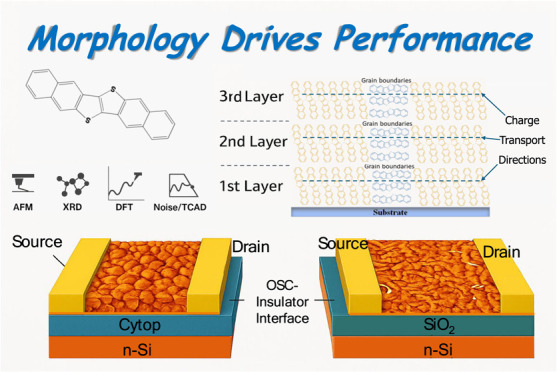

## 1 Introduction

Organic semiconductors are among the most studied materials for microelectronics. Thanks to their excellent physical and mechanical properties, their potential spans a large number of fields including electronic, optical, and biological applications, promising to be the strongest candidates to serve as the building blocks in a wide range of future electronic devices.^1,2^ These characteristics, combined with their easy handling, allow one to implement thin, flexible, and low-cost electronics, which can be produced using a sustainable, low-temperature process.^3,4^ In recent years, many devices based on small organic molecules have already been proposed and manufactured, such as organic thin-film transistors (OTFTs),^5–8^ light-emitting diodes (OLEDs),^9^ solar cells (OSCs),^10,11^ energy accumulators and batteries,^12^ chemical and mechanical sensors,^13,14^ photodetectors,^15,16^ biomedical sensing devices,^17^ and thermoelectric generators.^18,19^ Organic materials are therefore key drivers of the essential green and digital transition, not only to replace current amorphous silicon-based electronics but to be the basis of innovative devices.^20^ Among the most widely used and promising organic molecules, the p-type semiconductor dinaphtho [2,3-b:2′,3′-*f*] thieno [3,2-*b*] thiophene (DNTT) exhibits excellent transport properties combined with good environmental stability.^21–26^ DNTT films are typically deposited by sublimating the material in a vacuum chamber directly on the surface of a dielectric substrate. In most devices, particularly in OTFTs, the quality and properties of the interface between semiconductor and dielectric are of fundamental importance for their operation.^27–30^ The growth of the DNTT molecules on different dielectric substrates and their alignment in the first layers at the organic–dielectric interface have been recently intensively studied^31^ as their structure has a strong influence on the electrical properties of OTFTs.^30,32,33^ The long-standing debate about the arrangement of DNTT molecules on different substrates and the role played by the supramolecular orientation of the semiconductor material in the charge mobility is still a hot topic. It is widely reported that the best performances of the small-organic-molecule-based transistors, in terms of charge mobility as well as stability, are achieved when the molecules align with their long axis nearly perpendicular to the substrate. This arrangement promotes the highest overlap of the π–π orbitals and ensures efficient intermolecular charge transport along the substrate plane.^34^ In particular, the deposition of DNTT on silicon dioxide, SiO_2_, has been extensively studied due to its dielectric properties and low surface roughness. It has been reported that the DNTT molecules deposited on SiO_2_ align to form a homogeneous film, which is highly crystalline and exhibits good transport properties. In some cases, a layer of flat-lying ordered molecules has been reported.^33^ Similar behavior has not been seen on other substrates, although DNTT is grown on many of them.^24,35^ The role of horizontal orientation remains unclear, and the nature of the interfacial layer of flat-lying DNTT molecules on SiO_2_ is still debated.^24,32,33,36–38^ In this work, we investigate from both an experimental and a theoretical perspective the orientation of DNTT molecules at the semiconductor/dielectric interface in an OTFT architecture. In particular, the morphological and structural features of DNTT films with different thicknesses grown on SiO_2_ and an organic fluoropolymer (Cytop) were studied and combined with the computational studies about the structural and interaction energy and device’s electrical characteristics. The DNTT films were grown on SiO_2_ and on Cytop, in OTFT devices with a Bottom Gate Top Contact (BGTC) configuration (see the structure in Figure 1). Our main objective is to clarify the role of the substrate in determining the structure and morphology of DNTT films and to relate these factors to charge transport and device performance.

**Figure 1:**
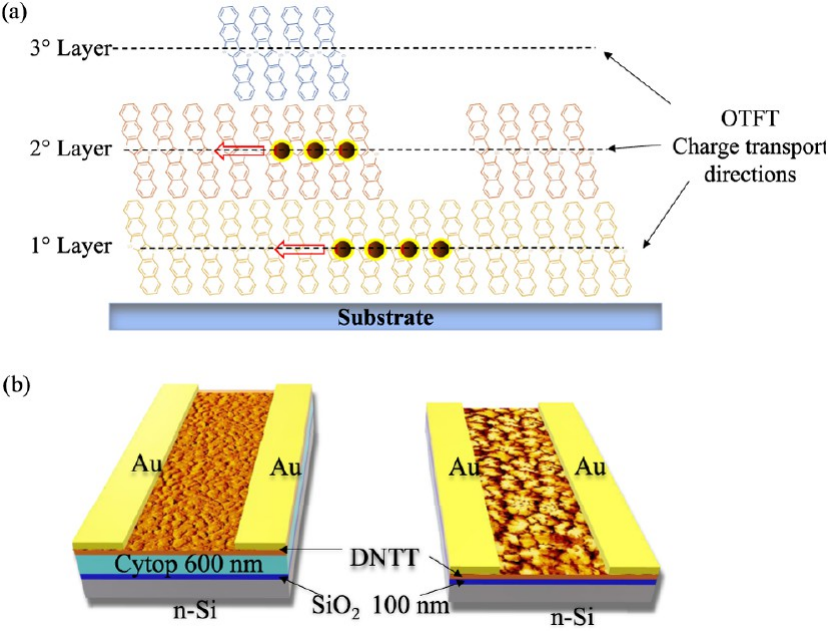
(a) Representative schematic illustration of the molecular packing in a thin film of DNTT. The dashed lines represent the directions of the electric charges (pictured by colored balls) in a typical transistor structure in which the channel is located parallel and adjacent to the substrate. (b) A schematic of OTFTs fabricated with a staggered Bottom Gate Top Contact (BGTC) structure. The two different configurations differ, from each other’s, in the nature of the substrate on which the DNTT thin film is grown. In one case (left) it is composed of a Cytop layer, 600 nm thick, while in the other case (right) it is composed of 100 nm of SiO_2_.

## 2 Results and Discussion

### 2.1 Electrical Characterization

Figure 2 shows the transfer characteristics of two representative OTFTs (channel length *L* = 500 μm, width *W* = 1 mm) in which the semiconductor layer (thickness: 50 nm) was grown on SiO_2_ (Figure 2a) and Cytop (Figure 2b), respectively. The electrical measurements were performed by keeping the source–drain voltage *V*_DS_ constant at −1 V, corresponding to the transistor’s linear operating region, while sweeping the source–gate voltage *V*_GS_ from 10 to −40 V and back from −40 to 10 V, as indicated by the black arrows in the graph. The *V*_DS_ value has been set to −1 V to ensure an appreciable drain current, even when the devices are biased in off conditions, while keeping the channel as uniform as possible, with negligible differences of carrier density between the drain and source ends. The curves are plotted on a linear scale (left axis) and a semilogarithmic scale (right axis). The field-effect mobility (μ_FE_), subthreshold slope (SS), and threshold voltage (*V*_TH_) values, extracted from these measurements,^39,40^ are reported in Table 1. The estimation of μ_FE_ in a linear regime was performed using the relationship

1 $$\mu_{\mathrm{FE}}=\frac{L}{C_{\mathrm{ins}}W_{\mathrm{eff}}V_{\mathrm{DS}}}\cdot \frac{\partial I_{d}}{\partial V_{\mathrm{gs}}}$$

evaluated at the maximum of the transconductance. In this equation, *C*_ins_ is the insulator capacitance per unit area, reported in Table 1, and *W*_eff_ (equal to 1.8 mm for a device with *L* = 500 μm and *W* = 1 mm) accounts for the increase of effective channel width, with respect to the nominal channel width *W*, that must be considered for device layouts in which the semiconductor layer has not been patterned and a significant amount of current flows in regions outside the nominal channel area.^41^ The field-effect mobilities evaluated from the transfer characteristics in saturation conditions (shown in Figure S1) are essentially identical to the μ_FE_ values evaluated in the linear regime. Process variability was also analyzed,^42^ and an evaluation of the associated errors is included in the table. The corresponding output characteristics of the devices are shown in Figure 2c,2d for DNTT/SiO_2_ and DNTT/Cytop OTFTs, respectively.

**Figure 2:**
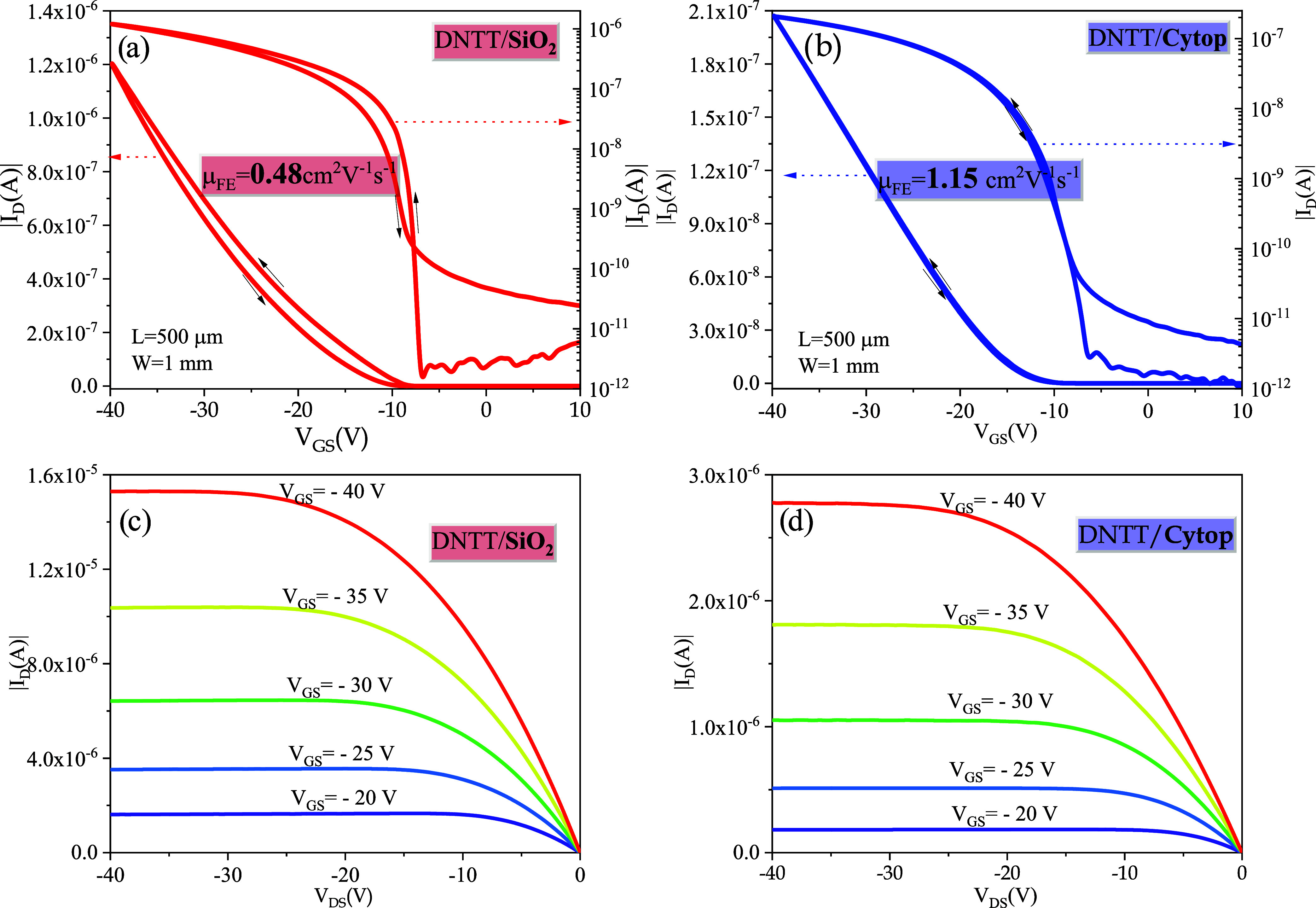
Up–down transfer characteristics for *V*_DS_= −1 V of (a) DNTT/SiO_2_ and (b) DNTT/Cytop OTFT devices with *L* = 500 μm and *W* = 1 mm. The measurements have been performed sweeping the *V*_G_ sequentially (ramp rate 1 V/s), from off-to-on (onward scan, from +10 to – 40 V) and from on-to-off (backward scan, from −40 V to +10 V) as indicated by the arrows in the graph; (c, d) corresponding output *I*_D_ vs *V*_DS_ characteristics (ramp rate 10 V/s) of DNTT/SiO_2_ and DNTT/Cytop OTFT devices, respectively.

**Table 1:** OTFT Device Parameters Calculated from the Measurements Reported in Figure 2a,2b

device	*C*_ins_ (nF/cm^2^)	μ (cm^2^/V· s) for *V*_DS_= −1 V	*g*_m_ (A/V·10^–8^) for *V*_DS_= −1 V	SS (V/dec)	*V*_TH_ (V)	hysteresis (*V*) for *I*_d_ = 1·10^–8^ A
**DNTT/SiO**_**2**_	35	0.48 ± 0.05	(3.45 ± 0.05)	0.3 ± 0.1	–22.0 ± 0.2	1.7 ± 0.1
**DNTT/Cytop**	2.8	1.15 ± 0.05	(0.44 ± 0.05)	1.4 ± 0.1	–16.0 ± 0.2	0.4 ± 0.1

The electrical characteristics show excellent OTFT performances, among the best currently reported for DNTT-based field-effect transistors.^24,43,44^ Hysteresis was also evaluated and the values calculated at fixed current are also reported in Table 1, indeed water and/or oxygen diffusion typically occurs in organic semiconductor-based devices which are still not properly encapsulated.^42,45,46^ Moreover, the output characteristics exhibit good linearity at low V_DS_, flat saturation zone, and absence of contact effects. This applies to both structures, although the mobility and SS of DNTT/Cytop devices are higher than the ones of DNTT/SiO_2_ devices.

To complement the conventional current–voltage measurements, the low-frequency noise analysis has been carried out on the fabricated OTFT by the high-sensitivity noise measurement system reported in ref 55. Measured noise spectra show a typical flicker (1/*f*)-like behavior and are interpreted in the context of the correlated mobility fluctuation (CMF) model.^47,56^

2 $$\begin{array}{ll} \sqrt{\frac{S_{\mathrm{ID}}}{I_{D}^{2}}}=\sqrt{k_{\mathrm{NF}}N_{t}}{[\frac{{-g}_{m}}{I_{D}}-\alpha \mu_{\mathrm{eff}}C_{\mathrm{ox}}]}^{2} & k_{\mathrm{NF}}=\frac{\mathrm{kT}q^{2}}{\mathrm{WL}C_{\mathrm{ox}}^{2}f} \end{array}$$

where *S*_ID_ is PSD of the current (*I*_D_) fluctuations, *f* is the frequency, *W* and *L* are the device channel width and length, *k* is the Boltzmann constant, *T* is the absolute temperature, *q* is the elementary charge, *C*_ox_ is the oxide capacitance per unit area, *g*_m_ is the transconductance, μ_eff_ is the effective mobility, α is the Coulomb scattering coefficient, and *N*_T_ is the interfacial trap density (cm^**–**2^eV^**–**1^).

Figure 3a shows the normalized PSD, measured at *f* = 1 Hz and at different *V*_GS_, as a function of −*g*_m_/*I*_D_ (eq 2) for Cytop and SiO_2_ devices of different gate lengths (50 μm ÷ 500 μm) and *W* = 1000 μm, while Figure 3b shows the corresponding trap density *N*_T_, calculated by eq 2, as a function of the measured mobility. The results shown in Figure 3 indicate a clear correlation between lower mobility and higher trap density and a much lower (about 2 orders of magnitude) trap density (higher mobility) in the Cytop devices with respect to SiO_2_ devices.

**Figure 3:**
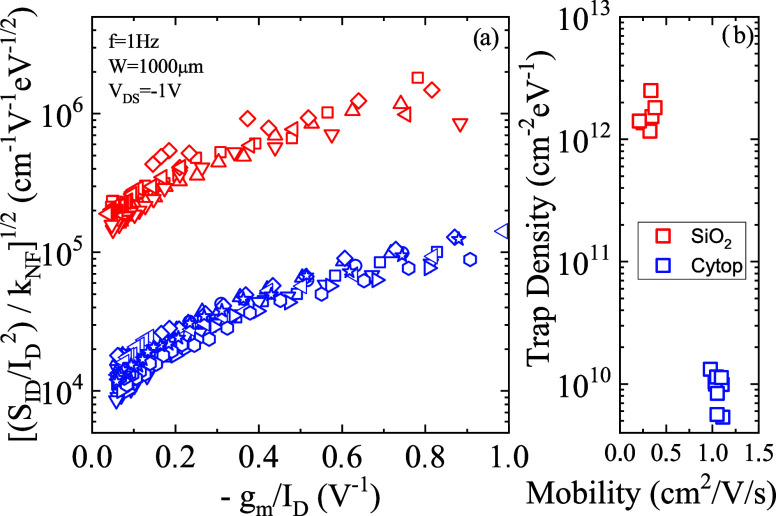
(a) Square root of the normalized noise PSD *S*_ID_/*I*_D_^2^ at *f* = 1 Hz, scaled by the geometrical factor *k*_NF_ defined in eq 2, as a function of the bias (−*g_m_*/*I*_D_) for Cytop and SiO_2_ devices of different gate lengths (50 μm ÷ 500 μm) and *W* = 1000 μm; (b) the corresponding trap density of individual devices, extrapolated by fitting the experimental data with the CMF model of eq 2, as a function of the effective mobility.

### 2.2 Atomic Force Microscopy (AFM) Characterization

The difference in the transport properties between these two devices, highlighted by the field-effect mobility and trap density measured by current–voltage and LFN measurements, leads us to study the morphology and structure of the semiconductor film grown on the two different dielectric materials.

Figure 4 shows the AFM images of the DNTT films, 50 nm thick, deposited on SiO_2_ (a–c) and on CYTOP (d–f). The AFM measurements were performed exactly on the films of OTFTs whose electrical characteristics are shown in Figure 2. DNTT films on the two dielectric substrates appear continuous but with a different morphology. The DNTT molecules on SiO_2_ are organized in grains of dendritic shape and lateral dimensions ranging in several microns. This aspect is often observed for small molecule organic films such as pentacene^8,34,48^ and DNTT^32^ and it is an indication of the high quality of the grown material.^49^ Figure 4b,c reports AFM images with large magnification, and the inset in Figure 4b shows the height profile corresponding to the blue line. The ramified DNTT islands on SiO_2_ show a layered structure with terraces (evidenced from the 3D topographic image in Figure 4c) hundreds of nm long and height of about 1.6 nm, which is in very close agreement with the length of a DNTT molecule.^50^

**Figure 4:**
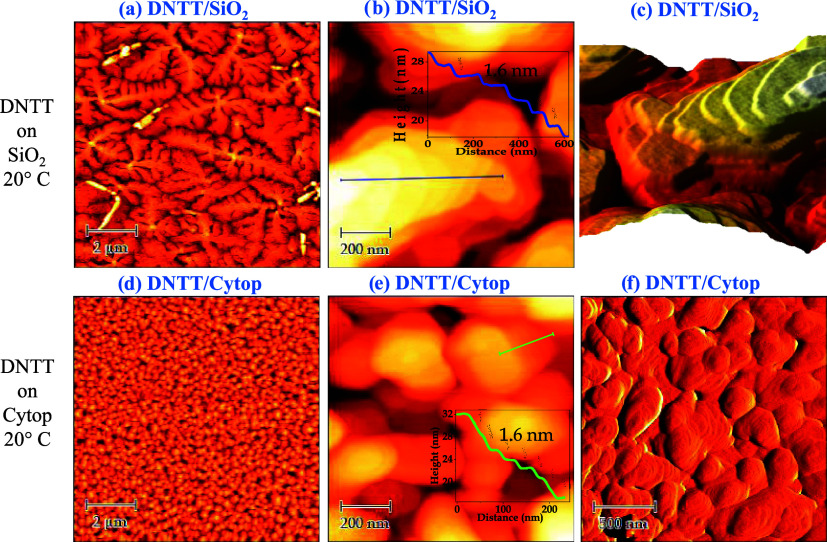
Morphological characterization of vacuum-deposited DNTT films of nominal thickness of 50 nm on SiO_2_ and Cytop substrates: (a, d) 10 μm × 10 μm, (b, e) 1 μm × 1 μm AFM topographic images, the insets in (b, e) are the height profiles corresponding to the solid lines; (c) a 3D 1 μm × 1 μm topographic AFM image corresponding to Figure 4b; (f) 2 μm × 2 μm error signal image of the DNTT/Cytop sample.

This points out that in all of the upper semiconductor layers visible by the AFM technique, the molecules are oriented upright with respect to the gate-dielectric surface. From this AFM analysis, SiO_2_ supports a correlated growth of semiconductor islands by the formation of polycrystalline DNTT films with high molecular order. The morphology of the DNTT film on Cytop appears very different from that of DNTT on SiO_2_ (see AFM in Figure 4d,4e). DNTT molecules arrange themselves in islands quite dimensionally regular, with an almost circular shape, a few hundred nanometers large, rather smaller than those of the film on SiO_2_. The islands appear connected, generating a homogeneous, compact film on a large scale. More in detail, a single island consists of a terrace structure with steps tens of nanometers large and about 1.6 nm high. The inset in Figure 4e shows the height profile, corresponding to the green line, which confirms a step height comparable to that observed for DNTT growth on SiO_2_. Figure 4f shows a 2 × 2 μm error signal image of the DNTT/Cytop sample that highlights the characteristic structure of the film composed of terraced coalescing grains. These results clearly indicate that the DNTT molecules, regardless of the substrate, arrange standing up on the dielectric surface, at least for the upper layers, and the polycrystalline DNTT thin films result to be highly textured.^32^ Although from the AFM analysis of the DNTT/SiO_2_ film the DNTT grain size is much larger compared with DNTT/Cytop film, the μ_FE_ is higher in DNTT/Cytop devices, as reported in Table 1 and Figure 2, indicating that some other characteristic of DNTT grown on SiO_2_ is limiting its charge transport properties. Since charge transport occurs in the very first DNTT layers growing from the substrate, it is important to evaluate the morphology of DNTT ultrathin films. To this purpose, AFM topographic images 5 μm x 5 μm of DNTT thin films with different thicknesses, deposited on SiO_2_ (top figures) and on Cytop (bottom figures), are shown in Figure 5. On SiO_2_, the DNTT molecules assemble from the beginning to form flat islands, with lateral sizes of several microns. The measured steps are all equal to the length of the molecule or its multiples, indicating a 2D-like growth already in the first levels. As the thickness increases, already at 6 nm, the grains start to connect, and a terraced structure is clearly prominent. The increasingly dendritic character of the branched grains becomes appreciable and dominant from 15 nm. Therefore, once the connection between all of the structures has been ensured, already at 15 nm, the morphology of the film appears isotropic with respect to the film thickness.

**Figure 5:**
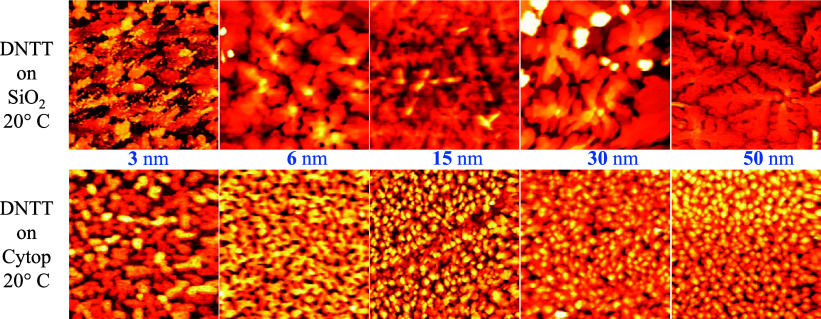
5 μm × 5 μm AFM topographic images of DNTT thin films deposited at room temperature (20 °C) on SiO_2_ (top figures) and on Cytop (bottom figures). The images refer to films with nominal thicknesses of 3, 6, 15, 30, and 50 nm (from left to right), respectively.

The formation of the film on Cytop is evidently different from just the first layers. The molecules deposited present less surface diffusion on the polymeric substrate forming smaller islands than those growing on SiO_2_. However, a terraced structure still holds with step heights equal to 1.6 nm, indicating that in all of the layers, from the beginning, the DNTT molecules arrange vertically with respect to the substrate. By increasing the number of molecules evaporated and then deposited, the grains remain round shaped, and they do not increase their diameter, which keeps a few hundreds of nm. With increasing thickness, the grains become increasingly connected and terraced, as well as for the film grown on SiO_2_, already at 15 nm the film appears well defined. Figure 6 shows the same information as Figure 5 in the case the substrate is maintained at 70 °C during the high vacuum evaporation of the DNTT film. This test is necessary to highlight any possible influence of the temperature on the growth of the DNTT films on the different substrates. We highlight that 70 °C is a process temperature considered compatible, well manageable, and accepted for organic electronics on almost all flexible substrates. When DNTT is evaporated on heated SiO_2_, the diffusion of the molecules on the surface increases. Consequently, the forming two-dimensional islands are much larger than those obtained at room temperature, already in the first stages of growth. The vertical molecular packing is verified by measuring the height of the steps of the islands, always being multiples of 1.6 nm. By increasing the lateral dimension of the grains, the connection between them increases as well. The dendritic, branched, and terraced character of the grains also remains under these growth conditions. The terraces have steps from hundreds of nanometers up to a few micrometers long for the first levels. On the contrary, when DNTT is deposited on Cytop at 70 °C, the growth assumes a three-dimensional behavior in the early stages of film formation. The layered grains formed are larger and taller than the ones grown at room temperature, with a less rounded shape and sharper edges, but they do not have a dendritic character and they are also not well connected to each other’s.

**Figure 6:**
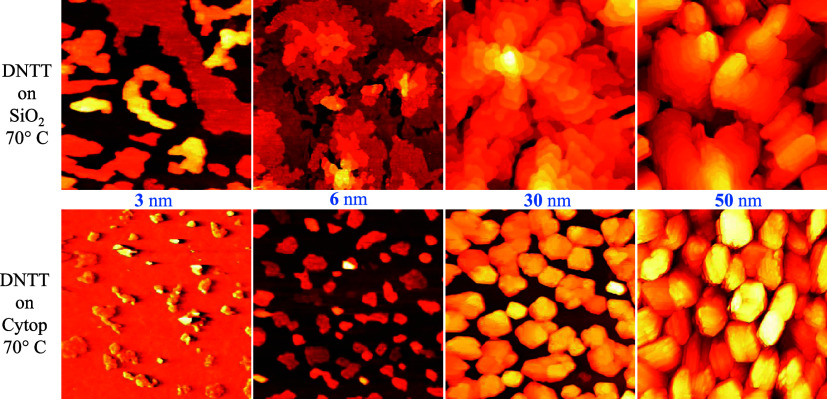
5 μm × 5 μm AFM topographic images of DNTT thin films deposited keeping the substrate at 70 °C on SiO_2_, top figures, and on Cytop, bottom figures. The images refer to films with nominal thickness of 3, 6, 30, and 50 nm (from left to right).

The mentioned tests clearly demonstrate that the formation of the DNTT evaporated film is strongly influenced by the substrate nature as well as the temperature conditions during deposition. More images and considerations are reported in Figure S2.

### 2.3 X-Ray Diffraction (XRD) Characterization

Deeper insight into DNTT molecular arrangement has been obtained by combining XRD measurements performed in specular out-of-plane (OP) and in-plane (IP) configurations, which are able to detect diffracted signals from crystalline planes lying parallel and perpendicular to the substrate, respectively. Figure 7a shows high-resolution OP-XRD profiles of the DNTT films (thickness 50 nm) deposited on SiO_2_ (red curve) and on Cytop (blue curve) substrates deposited at 20 °C. Both profiles present sharp peaks which can be indexed as (00l) reflections of the bulk crystal phase, up to the fifth order in the measured angular range.^24,50,51^ An insight with a higher resolution of the (003) peak is shown and analyzed in Figure S3.

**Figure 7:**
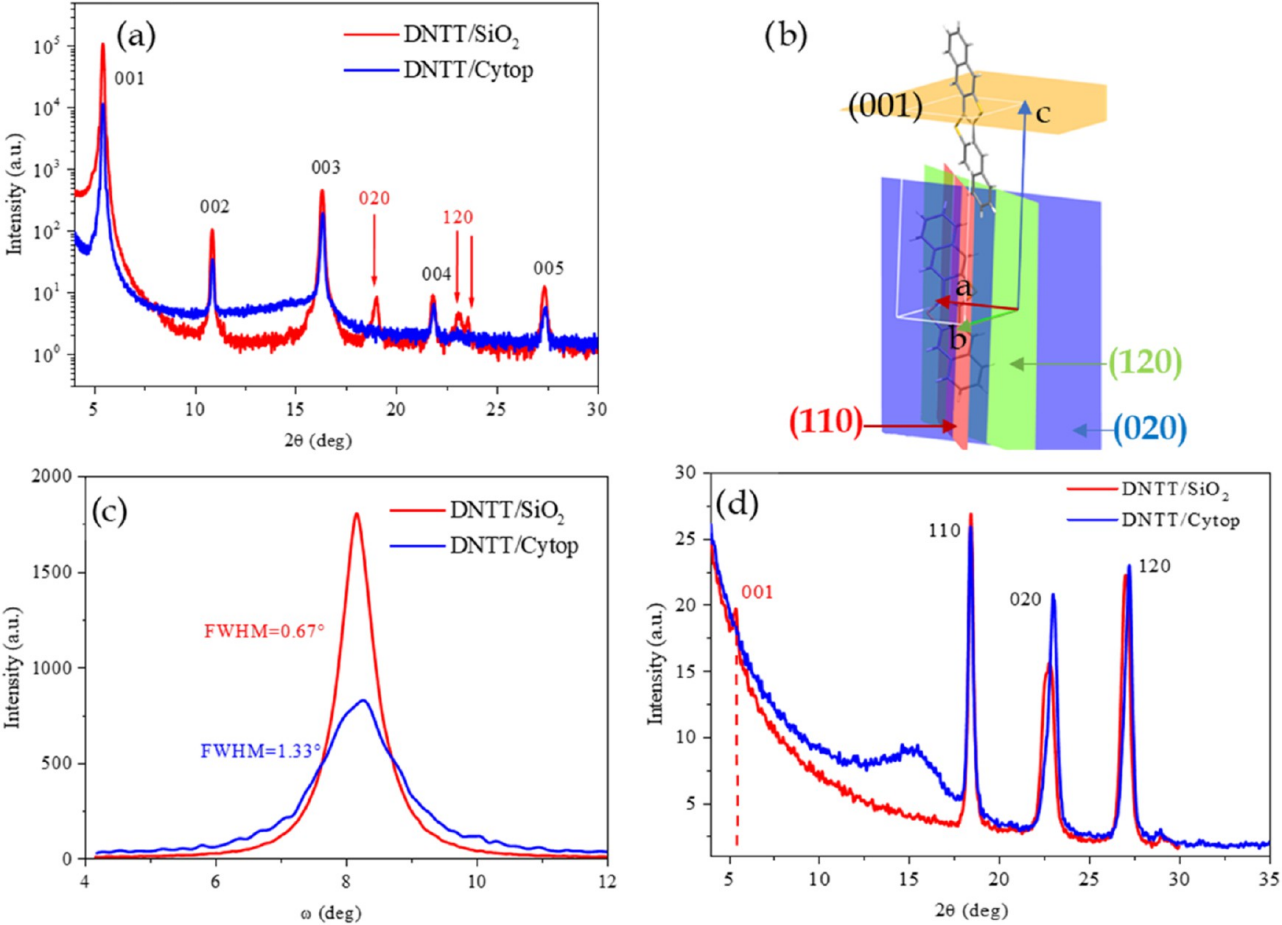
(a) High-resolution OP-XRD patterns; (b) a sketch of DNTT orientation on film structure; (c) (003) rocking curves; (d) IP-GIXD patterns of DNTT/SiO_2_ (red curve) and DNTT/Cytop (blue curve), respectively. The broad peak in (d) centered around 15° corresponds to the Cytop substrate.

This indicates that both films consist of crystallites highly oriented with (00l) parallel to the substrate surface, i.e., with the long molecular axis almost perpendicular to it (see sketch reported in Figure 7b). The (001) *d* spacing is 1.6 nm, in good agreement with the step height determined by AFM measurements. From FWHM of (003) diffraction peak, using the Debye–Scherrer equation,^52^ the vertical coherence length of the crystalline domains has been estimated to be close to the nominal film thickness (59 and 63 nm, respectively, for the film on SiO_2_ and Cytop). Figure 7c shows the rocking curves for the (003) reflection whose HWHM is a measure of the misorientation degree of the crystalline domain, called mosaicity, with respect to a perfect parallelism with the film surface. The mosaicity of DNTT/Cytop (0.67 deg) is slightly larger than that of DNTT/SiO_2_ (0.34 deg), corresponding to a larger spreading of the DNTT molecules orientation with respect to the perfect edge-on configuration. The lower peak intensity values of the DNTT/Cytop film are compatible with this wider misorientation, although structural effects, such as modified static Debye–Waller factors, which would point to disorder or significantly enhanced molecular mobility, are not precluded. However, the biggest difference between the two specular profiles consists in the presence, only for the film on SiO_2_ of two additional peaks (marked by arrows in Figure 7a), corresponding to the (020) and (120) planes. This proves the presence of ordered molecules having a long axis parallel to the substrate. The IP-grazing incidence X-ray diffraction (IP-GIXD) measurements reported in Figure 7d confirm this result. Both profiles show three strong peaks, indexed as the (110), (020), and (120) reflections of the bulk phase. The quite pronounced peak intensity indicates a good DNTT packing film in the surface direction. The horizontal crystallite domain is estimated by the FWHM, *D* = 29 and 32 nm, on SiO_2_ and CYTOP, respectively. The weak (001) peak recorded for the film on SiO_2_ can be explained by a small scattering volume, i.e., few crystallites oriented so that DNTT molecules lie with the long axis parallel to the substrate.

To investigate in more detail the structural organization, molecular orientation, and degree of crystallinity within the active layer, we performed grazing incidence wide angle X-ray scattering (GIWAXS) measurements. This technique is particularly well suited for probing thin films, as it combines surface sensitivity with the ability to resolve in-plane and out-of-plane ordering in nanostructured materials. The resulting diffraction patterns provide detailed insights into the crystalline texture and preferential orientation of molecular domains, which are critical parameters influencing charge transport and device performance. GIWAXS patterns of DNTT films acquired with an incident angle of 0.1° using an X-ray wavelength of 1 Å have been reported in Figure 8. For thinner films (3 nm), the (00l) texturing is highlighted both on SiO_2_ and on Cytop substrates, demonstrating that since the initial layers, the DNTT molecules mainly stand up on both substrates, as previously shown by the AFM and HRXRD measurements. The more circular shape and the greater intensity of the spots for the DNTT film on SiO_2_ show a greater order of the vertical stack, right from the first closest layers to the substrate. Increasing the thickness, in the DNTT/SiO_2_ films, diffraction signals from differently oriented DNTT molecules merge, i.e., along the (0k0) and (hk0) directions as previously shown in Figure 6a and confirmed by the simultaneous presence of (00l), (0k0), and (hk0) texturing revealed in Figure 8b. In DNTT/SiO_2_ structures, DNTT horizontal crystallite patterns are not present in the thinnest film but start to appear when the film is 15 nm thick, reaching the maximum intensity for the highest thickness. This proves that the horizontal crystallites are found along the entire thickness of the DNTT film and not only at the SiO_2_ interface. However, for 3 nm samples, the signal coming from them is very weak, coming from a very small portion of horizontal crystalline film, and is therefore indistinguishable from the experimental measured noise. GIWAXS images of intermediate thickness films are reported in S3 (Figure S10). Moreover, the invariance of the 2D-GIWAXS images recorded at different incidence angles (from 0.05° to 0.1°), reported in Figure S11 of Section S3 indicates that the same molecular arrangement is maintained along the film thickness. The diffraction signals related to molecules lying flat on the SiO_2_ surface, although weak, certainly come from crystallites and not from a monolayer. The strengthening of these signals with increasing film thickness indicates that these crystallites are distributed along the entire thickness of the film.

**Figure 8:**
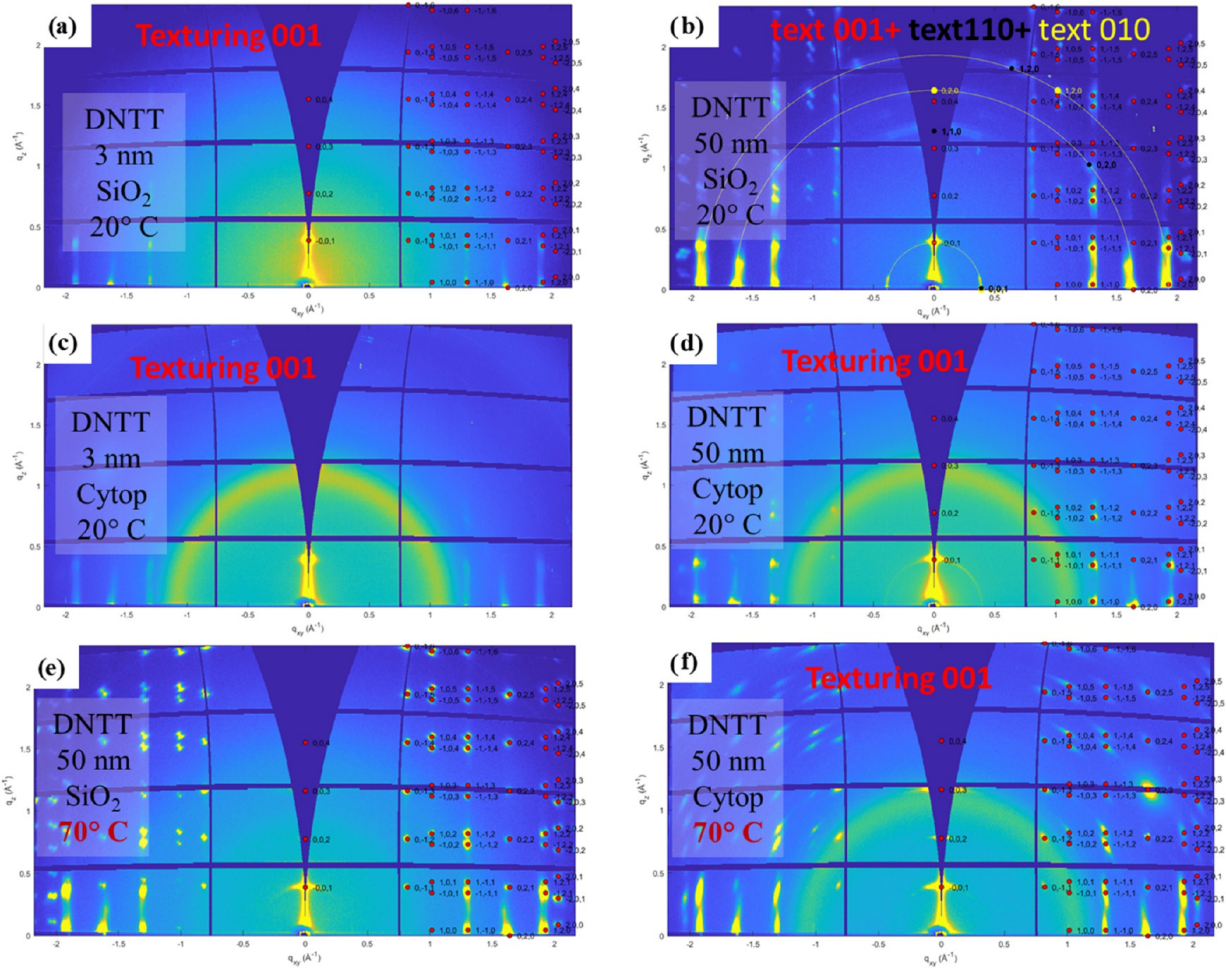
2D-GIWAXS images of DNTT films: (a) DNTT/SiO_2_ 3 nm deposited at 20 °C; (b) DNTT/SiO_2_ 50 nm deposited at 20 °C; (c) DNTT/Cytop 3 nm deposited at 20 °C; (d) DNTT/Cytop 50 nm deposited at 20 °C; (e) DNTT/SiO_2_ 50 nm deposited at 70 °C; (f) DNTT/Cytop 50 nm deposited at 70 °C. More GIWAXS of these films are reported in the Supporting Information (Figures S10 and S11).

When DNTT is deposited on SiO_2_ heated to 70 °C, only the vertical texturing (00l), i.e., edge-on molecular orientation, is present, as shown in Figure 8e, and in HRXRD reported in Figures S4 and S5. For all of the DNTT/Cytop films (Figure 8c,d,f), the only measured texture is the (00l), i.e., vertical molecular stacking. Moreover, these diffraction peaks are less intense and have fewer ideal shapes compared with those of the DNTT/SiO_2_ films, confirming what is reported in Figure 7c. Therefore, the molecules oriented along the (00l) in the DNTT/SiO_2_ films are more ordered than the counterpart in the DNTT/Cytop films which, however, do not present any crystallite with horizontally oriented DNTT (Figures S7 and S8). Moreover, DNTT/Cytop films, unlike those on SiO_2_, if deposited at higher temperatures (70 °C, Figure 8f) increase their mosaicity in the direction (00l), confirmed by RC reported in Figures S6 and S9.

The results of the detailed morphological AFM and XRD analysis can be explained with the presence of crystallites of flat molecules on SiO_2_, deposited at 20 °C, along the thickness of the DNTT films, and not only at the interfaces with the dielectric as previously reported.^33^

### 2.4 Ab Initio Density Functional Theory (DFT) Calculation

To theoretically examine the growth of DNTT on the SiO_2_ surface and to understand the molecule–surface interactions involved, we investigated the changes in adsorption geometry and the interaction of DNTT with the SiO_2_ surface, using density functional theory (DFT) calculations. In analogy to a previous study^36^ that demonstrated stabilized adsorption and orientation changes for pentacene on SiO_2_, we aimed to determine the preferred DNTT orientation, guided by experimental data suggesting a vertical alignment. Our DFT calculations clarify the specific energetic contributions driving the orientation of DNTT on SiO_2_, accounting for both single-molecule interactions and subsequent layer stabilization, as quantified through adsorption energy decomposition. To this end, we analyzed two potential orientations of the DNTT molecules: vertical and horizontal to the SiO_2_ surface^37,53^ examining their respective interactions. We initially focused on isolated DNTT molecules, aiming to simulate the preliminary phase of crystal formation. The interactions of these molecules with the SiO_2_ surface were quantified by evaluating the adsorption energies (*E*_ads_) and interaction energies (*E*_int_) using the definitions outlined in the methods section. Given the amorphous nature of SiO_2_ and the presence of OH groups irregularly distributed on the surface, we initially identified the most favorable adsorption sites by evaluating several positions (see Figure S12). Six possible adsorption sites, including isolated and vicinal OH groups, were considered for each molecular orientation and labeled V1–V6 for vertical and H1–H6 for horizontal orientations. In Figure S12c, a comparison of the adsorption energies for DNTT at these six positions is provided. It is seen that horizontal orientation is generally more favorable than vertical orientation during the early stages of film growth. In particular, structures V3 and H5 exhibit lower *E*_ads_ values, indicating that these sites provide a more energetically favorable environment for DNTT adsorption (Figure S12d). Comparing adsorption configurations V3 and H5, it is seen that the horizontal orientation is more favorably adsorbed on SiO_2_ than the vertical orientation. This is suggested by the lower *E*_ads_ of −1.01 eV in the horizontal geometry H5, compared with −0.44 eV for the vertical one, as shown in Table 2. With the aromatic core of the DNTT molecule lying flat, it can maximize favorable π-surface contacts with the electron density of the surface and strengthen van der Waals (vdW) forces at the interface. Despite greater structural distortion, the enhanced surface interaction leads to a higher overall stability. This conclusion is further supported by the more negative *E*_int_ of −1.32 eV in the horizontal configuration, reflecting stronger combined electrostatic and vdW interactions, compared with – 0.55 eV in the vertical configuration. Variations in charge density provide further insights into the electronic properties of the adsorbed molecule and its interaction with the SiO_2_ surface. In the vertical orientation (Figure 9a,c), the proximity of the molecule to the surface results in O–H···C interactions involving the π-system of the double bonds, supported by additional C–H···O hydrogen bonds. In the horizontal orientation (Figure 9b,d), similar interactions occur across a larger molecular contact area, supplemented by interactions where surface hydroxyl groups interact directly with the aromatic π-electron clouds, forming stabilizing O–H···π interactions. Electron density redistribution, indicated by regions of electron accumulation (yellow) and depletion (cyan), highlights these interactions clearly in both orientations.

**Figure 9:**
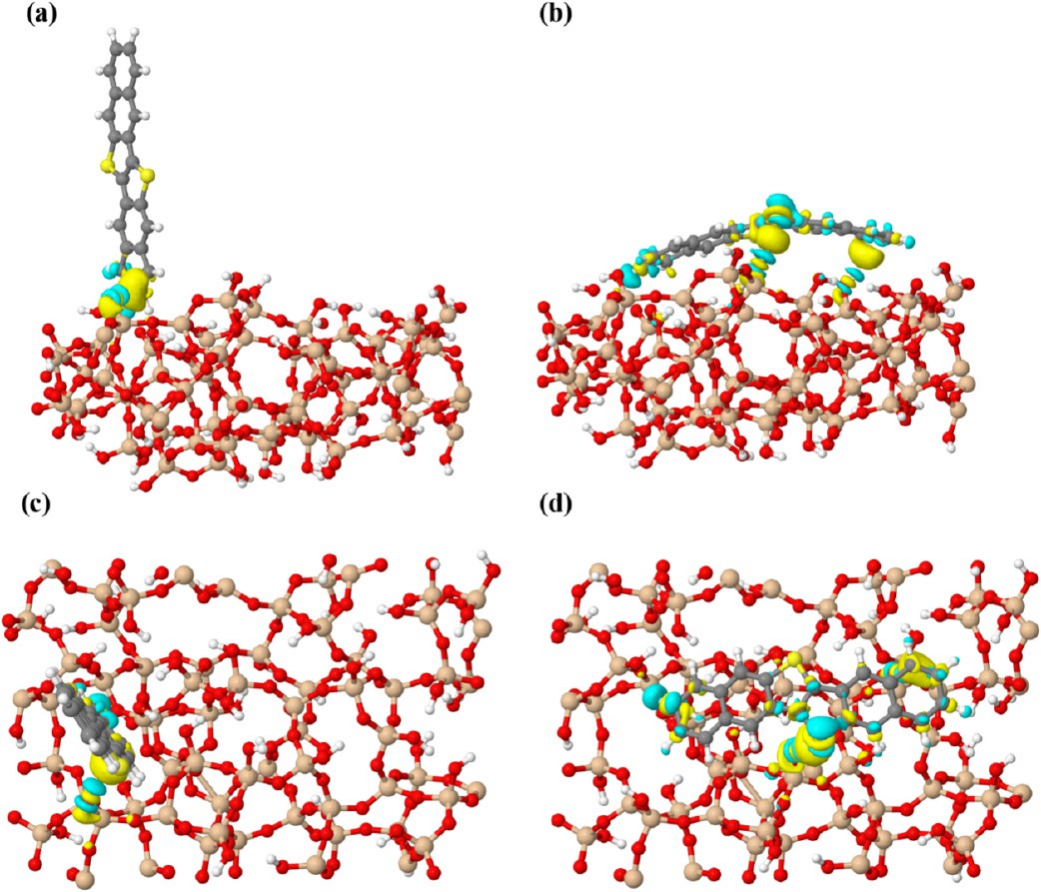
Charge density difference for a DNTT molecule adsorbed on the SiO_2_ surface, where yellow and cyan areas indicate electron accumulation and depletion, respectively (the isosurface is 6 × 10^**–**3^ eÅ^**–**3^). (a) Side view and (c) top view for the vertical orientation; (b) side view and (d) top view for the horizontal orientation.

**Table 2:** Energetic Characteristics of DNTT and a Layer of DNTT Adsorbed on SiO_2_ ^a^

structures	*E*_ads_	*E*_int_	*E*_*D3*_	*E*_bind_(D)
V3	–0.44	–0.55	–0.35	0
H5	–1.01	–1.32	–0.97	0
Full_V	–1.41	–1.51	–1.59	–1.38
Full_H	–1.34	–1.39	–1.52	–1.21

a Adsorption energy (*E*_ads_), interaction energy (*E*_int_), dispersion energy (*E*_D3_), and the molecule–molecule interaction (*E*_bind_(D)). All energies presented in the table are given in eV per molecule.

Therefore, the favorable charge distribution in the case of the horizontal orientation suggests an enhanced electron delocalization over the molecule–surface interface, which could be responsible for the increased stabilization. In the second stage, we increased the number of molecules on the surface while maintaining the same orientations (vertical and horizontal). Figure 10 illustrates the DNTT layer consisting of 16 molecules on the amorphous SiO_2_ surface. This stage aimed to predict the energies associated with the formation of the semiconductor layer as additional DNTT molecules are introduced. Under these conditions, a larger number of organic molecules can potentially cover the entire substrate, requiring careful consideration of intermolecular interactions. The adsorption behavior changes significantly when a layer of DNTT molecules is considered. From Table 2, the *E*_ads_ for a single molecule in the vertical orientation is −0.44, and −1.01 eV in the horizontal one. However, for 16 molecules, it is −1.41 eV vertically and −1.34 eV horizontally. This increase suggests that the molecules stabilize each other when in close proximity, reducing the energy gain from adsorption on the surface. This highlights the significance of intermolecular interactions at high coverage. In these structures, the molecular–molecule interaction energy (*E*_bind_(D)) becomes strongly negative (−1.38 eV), indicating intensified intermolecular forces. Additionally, the energy dispersion (*E*_D3_) in vertically oriented molecules surpasses that in horizontal orientation, which can compensate for the lower molecule–surface contact area in the vertical orientation. In a dense molecular layer, these intermolecular forces become more influential than the individual molecule–surface interactions that dominate in the case of a single, isolated molecule. Therefore, as the crystal begins to grow, stronger intermolecular interaction, particularly enhanced π–π interaction, becomes more pronounced.^38^ As a result, while an isolated DNTT molecule lying flat exhibits favorable adsorption due to surface interactions, the vertical orientation becomes more energetically favorable as the number of molecules increases. We further examined this behavior by decomposing the adsorption energy into several components, including surface and molecular deformation. This analysis showed that the horizontal orientation imposes a greater penalty through substrate deformation, while the vertical orientation is favored due to lower surface deformation, improved formation energy, and stronger molecule–molecule interactions (more details are given in Section S4). This agrees with experimental observations, where the vertical orientation of DNTT is commonly seen in islands formed on SiO_2_ surface. A similar behavior was also observed in DNTT films analyzed by X-ray diffraction on pristine KCl, yielding a (001)-oriented crystalline layer.^54^

**Figure 10:**
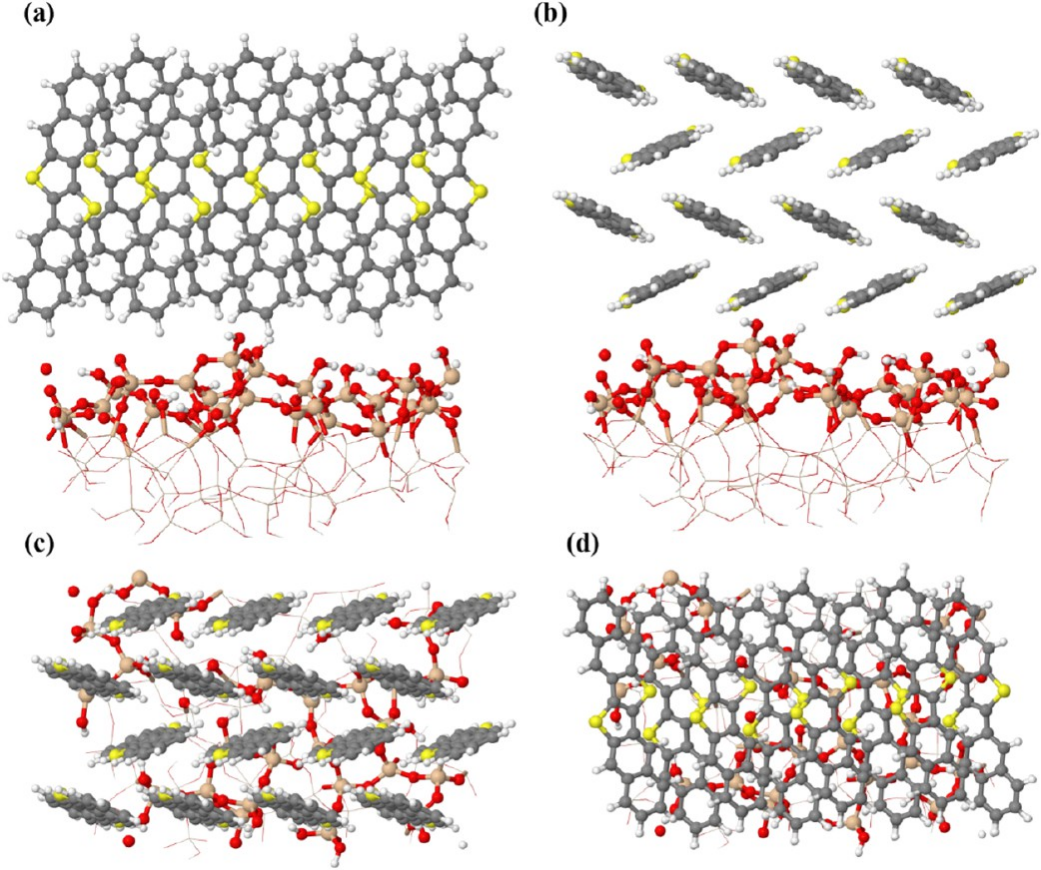
Top and side views of the DNTT molecules adsorbed on the SiO_2_ surface: (a) side and (c) top view in the vertical orientation, and (b) side and (d) top view in the horizontal orientation. For better visualization, the lower atoms and bonds are displayed in the wireframe.

### 2.5 Connective Tissue between DNTT Grains

AFM, XRD measurements, and ab initio simulations confirm that the DNTT film grows with a vertical orientation from the first levels in contact with the substrate. Therefore, it is plausible to assume that the horizontal DNTT crystallites present in DNTT/SiO_2_ films are located at the grain boundaries of the highly vertical orientated dendritic structures of DNTT on SiO_2_. We hypothesize that the connective tissue between the grains is formed by oriented molecules that can negatively affect the transport of charges between grains, creating areas with different conductivity. Similar behavior has been very recently reported for thienoacene-based organic semiconductor, 2,9-diphenyldinaphtho­[2,3-b:2′,3′-*f*]­thieno­[3,2-*b*] thiophene (DPh-DNTT) thin films deposited on metallic ITO substrates.^55^ In Figure 11, the transfer characteristics of OTFT DNTT/SiO_2_ devices realized keeping SiO_2_ at 20 and 70 °C are shown for DNTT film with thickness of 6 nm (Figure 11a) and 50 nm (Figure 11b). When increasing the SiO_2_ temperature to 70 °C during deposition, horizontally oriented crystallites do not grow, as shown by XRD measurements, and the electrical characteristics and the mobility of the sample improve. Moreover, as shown in AFM images, for the 6 nm thick film, the connection between grains was improved from the very first levels on DNTT vertical oriented molecules. On Cytop, on the other hand, the DNTT molecules prefer to stack always in a vertical position, and the correct alignment of the DNTT molecular orbitals is always guaranteed, making the mobility higher for DNTT/Cytop devices than DNTT/SiO_2_ devices.

**Figure 11:**
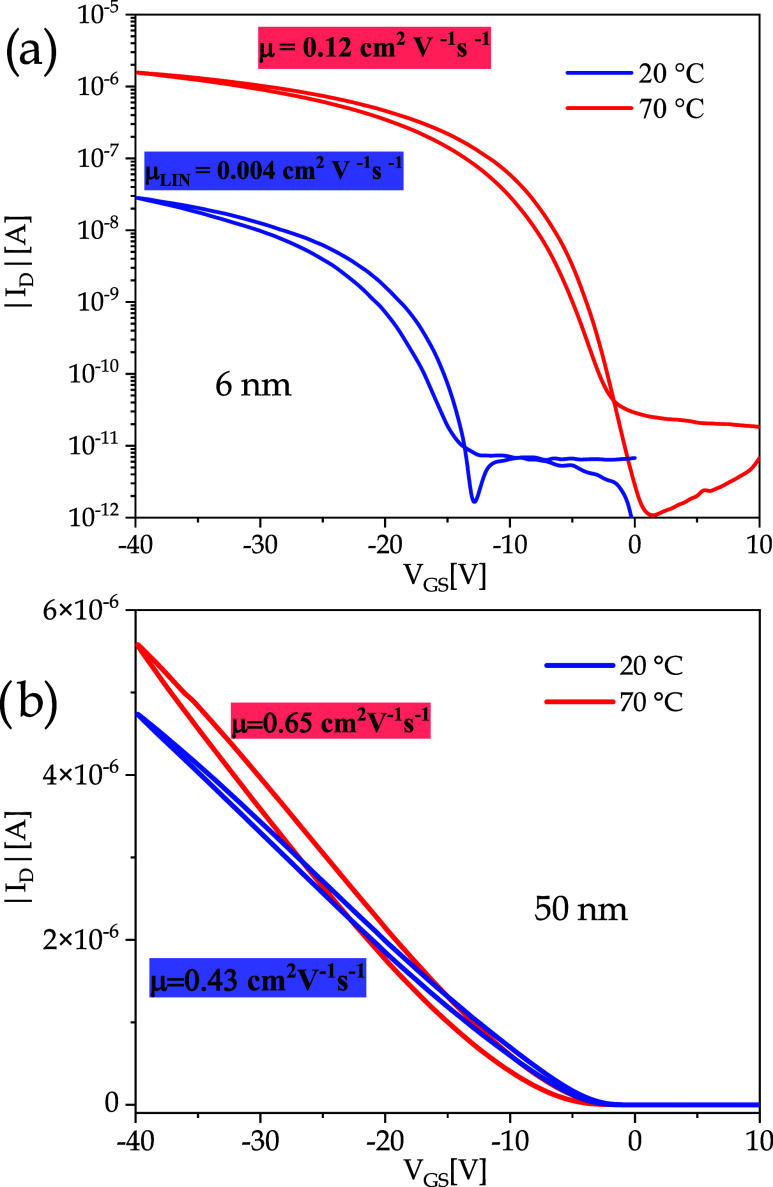
Up–down transfer characteristics for *V*_DS_= −1 V of DNTT/SiO_2_ OTFT devices with DNTT film thickness of (a) 6 nm and (b) 50 nm. The blue and red curves refer to film grown keeping SiO_2_ at 20° and 70 °C, respectively.

### 2.6 TCAD Simulation

The electrical transfer characteristics of OTFTs fabricated with 50 nm thick DNTT films grown on CyTop at 20 °C and on SiO_2_ at 20 and 70 °C have been reproduced using the 2D numerical device simulation software “Sentaurus” provided by Synopsys Inc. as a part of the “Synopsys TCAD” software package.^56^ It has been shown that transport in organic materials can be effectively simulated using TCAD programs that solve the drift-diffusion equations choosing an appropriate value for the carrier band mobility and introducing suitable trap states distributions in the HOMO–LUMO energy gap.^57^

Following this approach, the characteristics of OTFTs with DNTT grown on Cytop at 20 °C have been accurately reproduced, using a quasi-static simulation approach, in both linear and subthreshold regime (simulation A, see Figure 12a, light blue curves) using as a DOS an exponential tail of donor states with characteristic energy *E*_0_ = 84 meV (the light blue curve labeled “DOS-CyTop,” reported in Figure 12d) and a band mobility μ_DNTT/CyTop_ ≈ 1.7 cm^2^/(V s). In the case of OTFTs with DNTT grown on SiO_2_ at 70 °C, a good fit of the transfer characteristics, shown in Figure 12b (“simulation B,” green curves), was obtained using a slightly lower band mobility μ_DNTT/SiO2_ ≈ 1.1 cm^2^/(V s) and introducing a DOS described by a piecewise linear function (green curve “DOS-SiO_2_” in Figure 12d). A comparison with “DOS-CyTop” shows that DOS-SiO_2_ is a factor 50–120 times higher (compared with curve “DOS-CyTop x 100”), depending on the energy. Actually, the energy-integrated DOS in DNTT grown on the SiO_2_ case is equal to 4.52 × 10^19^ cm^**–**3^, 126 times larger than the energy-integrated DOS in DNTT grown on CyTop case, which is equal to 3.57 × 10^17^ cm^**–**3^. This confirms the presence of a higher trap density in SiO_2_-based devices with respect to the CyTop-based device, which has been evidenced also by noise analysis (see paragraph 1) as well as the correlation between higher DOS and lower carrier mobility.

**Figure 12:**
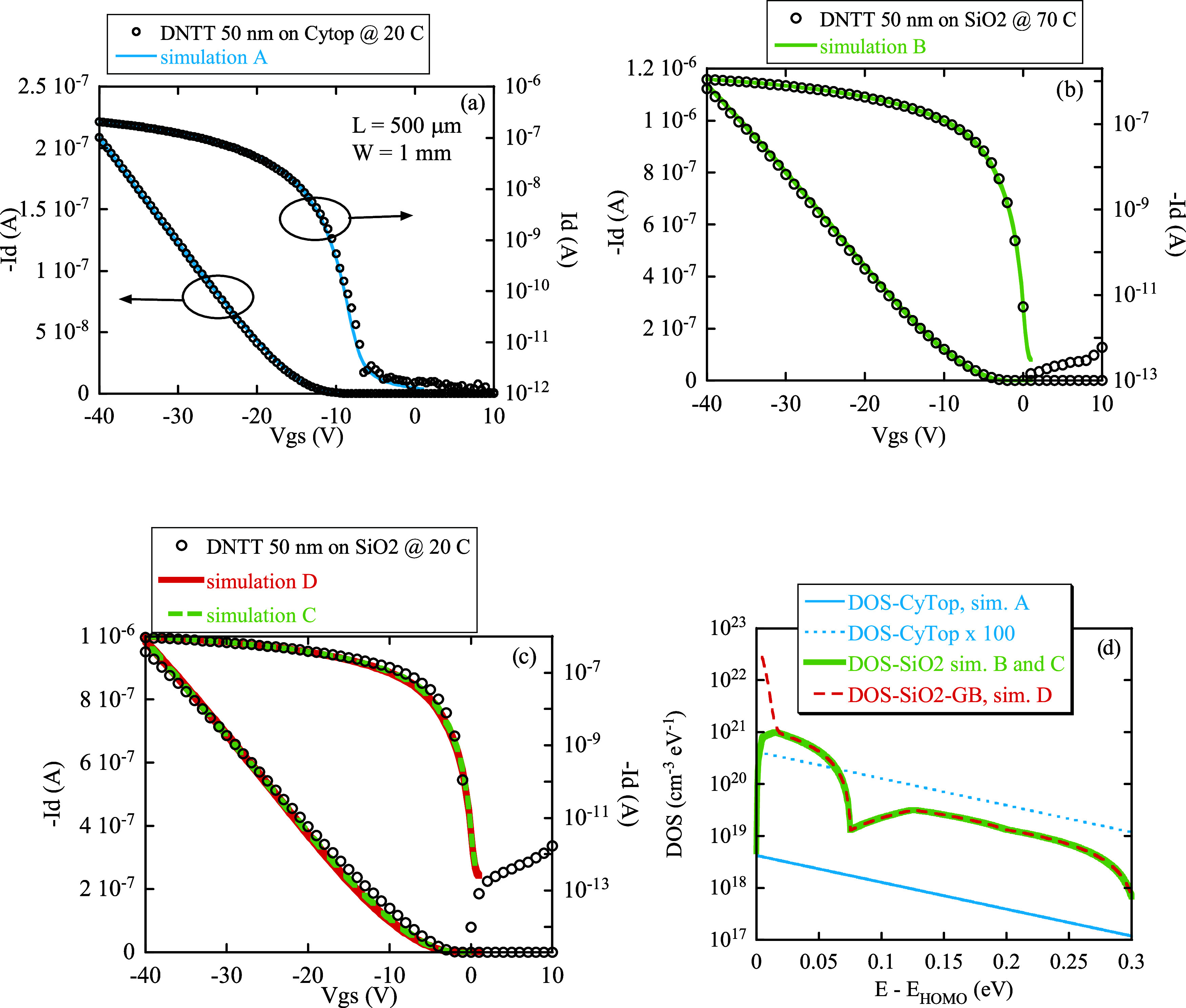
Numerical simulations (lines) of the OTFT transfer electrical characteristics compared with the experimental ones (symbols). (a) OTFT with DNTT grown on CyTop at 20 °C. (b) OTFT with DNTT grown on SiO_2_ at 70 °C. (c) OTFT with DNTT grown on SiO_2_ at 20 °C assuming a spatially homogeneous DOS (“simulation C”) or an enhanced DOS in the grain boundaries (“simulation D”). (d) Corresponding DOS used in the simulations shown in panels (a–c): for ease of comparison, the colors of the curves correspond to the colors of the simulated electrical characteristics shown in panels (a–c). The curve “DOS-CyTop x 100” is just a reference for comparison with the curve “DOS-SiO_2_.” The thickness of the semiconductor layer is 50 nm in all cases.

In both the previous cases, DNTT grown on SiO_2_ at 70 °C and DNTT grown on CyTop, DOS used in the simulations has been assumed spatially uniform. To follow the indications of the XRD and DFT analysis, the assumption of spatial homogeneity was released in the simulations of OTFTs with DNTT grown on SiO_2_ at 20 °C: in this last case, the organic semiconductor film was modeled as a sequence of grains, 1 μm long, separated by grain boundary (GB) regions of 2 nm wide (see Figure S13). Then, we tried to check if the slight modifications of the transfer characteristics between the devices with DNTT grown on SiO_2_ at 20 and 70 °C, shown in Figure 11b, are compatible with the introduction of additional defects and/or a mobility reduction in GB.^58^ To this end, in the simulations of the devices with DNTT grown on SiO_2_ at 20 °C, DOS and the mobility inside the 1 μm long grains have been kept equal to the values obtained on OTFT with DNTT grown on SiO_2_ at 70 °C (the green curve “DOS-SiO_2_” in Figure 12d), while two different approaches have been used for DOS and mobility inside the 2 nm long GBs. In the first case, shown as “simulation C” in Figure 12c (the green curve), we reduced the mobility in GB to 0.01 cm^2^/(V s) while keeping GB DOS equal to DOS-SiO_2_ (the green dashed curve in Figure 12d). In the second case, labeled “simulation D” in Figure 12c (red curve), we used an enhanced density of states in the GB regions, adding a very high donor Gaussian peak near the HOMO level (red dashed curve “DOS-SiO_2_-GB” in Figure 12d) and at the same time we used a band mobility equal to 0.04 cm^2^/(V s). As can be seen, the two approaches give nearly identical results, and both compare very well with experimental measurements. Thus, 2D numerical simulations of the devices with DNTT grown on SiO_2_ at 20 °C are compatible with the presence of GBs in the active layer, introducing regions with reduced electrical conductance with respect to the bulk of the material but do not allow us to unambiguously determine the exact mobility and the corresponding density of states in GBs. Details and simulation parameters are given in S5.

### 2.7 Organic Phototransistor Behavior for Different Semiconductor/Dielectric Interface Devices

In the previous paragraphs, we have highlighted how the transistors realized, based on different dielectric/semiconductor interfaces, are characterized by important differences with respect to the field-effect mobility, as initially shown in Figure 2. But different interfaces also have important implications on the optoelectronic characteristics and performances of organic phototransistor (OPT) devices based on the SiO_2_/DNTT semiconductor dielectric structure. In Figure 13a, the realized 3D schematic of the OPT device is reported, while in Figure 13b is shown the picture of a DNTT/Cytop sample realized on a flexible substrate. These photodetectors have the same BGTC staggered structure of the devices shown in Figure 1 and the complete description of their fabrication and characterization is reported elsewhere.^42,59^ This device coupled with an organic scintillator is a proton detector giving a promising tool for real-time particle detection over a large area and irregular surfaces, suitable for many practical, experimental scientific research and innovative medical applications.^59,60^ OPT has an impressive low light detection, with a LoD down to 28 pW/cm^2^,^61^ working reliably in different environments, with low polarization bias. The operating principle of OPT can be summarized as follows. When an incident photon, at the energy fitting of the optical absorption peaks of the semiconductor, is absorbed, an exciton is generated. The exciton is separated thanks to the source–drain voltage which leads the hole to contribute to the drain current *I*_D_, being DNTT a p-type organic semiconductor, while the electron is trapped in trap states in the gap of the semiconductor. This trapping of electrons shifts the threshold voltage of the transistor. Consequently, the current at the drain electrode *I*_D_, under illumination, undergoes an increase proportional to the amount of photons absorbed which is defined as photocurrent *I*_ph_ = *I*_D_(light) – *I*_D_(dark). The decrease of *I*_ph_ to its initial value consists of recovering the dark condition, and the time taken for this purpose depends on the detrapping time of the photogenerated electron. The complete working mechanism and time performances of the OPT response under pulsed illumination, both formation and decay of the *I*_D_ vs *t* waveforms, can be reproduced exploiting a kinetic model presented in a previous work.^59^ In Figure 13c and 13d the dynamic photoresponse, *I*_D_ vs *t*, of DNTT/SiO_2_ and DNTT/Cytop OPT devices, respectively, is shown, under three 450 nm light pulses of 2 min at an incident power density *P*_opt_ = 2.6 nW/cm^2^. The yellow bars on the graph represent the time laps when the device is illuminated. The measurements are acquired in the air. Both detectors can reproducibly and sensibly read the light signal, but with notable differences in the response. The first aspect to highlight is that DNTT/Cytop OPT is more stable under bias stress than the DNTT/SiO_2_ one, in fact, the trend of the *I*_D_ (dark) current with respect to time at fixed polarizations (for many hours, as required in many applications) is constant for DNTT/Cytop while it increases for DNTT/SiO_2_. In fact, to have a device that is very sensitive to light, all other types of electrical instabilities must be minimized, from environmental ones to bias stress, for example. In this respect, the DNTT/Cytop framework is favored. Moreover, this device recovers the light very quickly after illumination unlike what the detector based on the DNTT/SiO_2_ junction does. This allows us to use the device in a more reproducible and frequent way with decreasing dead times. These considerations demonstrate that the trap states for the photogenerated electrons are probably deeper in energy in DNTT/SiO_2_. These differences in performances are also a consequence of the morphology of the film grown on different substrates and demonstrate that growing DNTT on Cytop not only allows to have flexible, full organic devices but also provides higher field-effect mobilities and better optoelectronic characteristics for photodetector applications.

**Figure 13:**
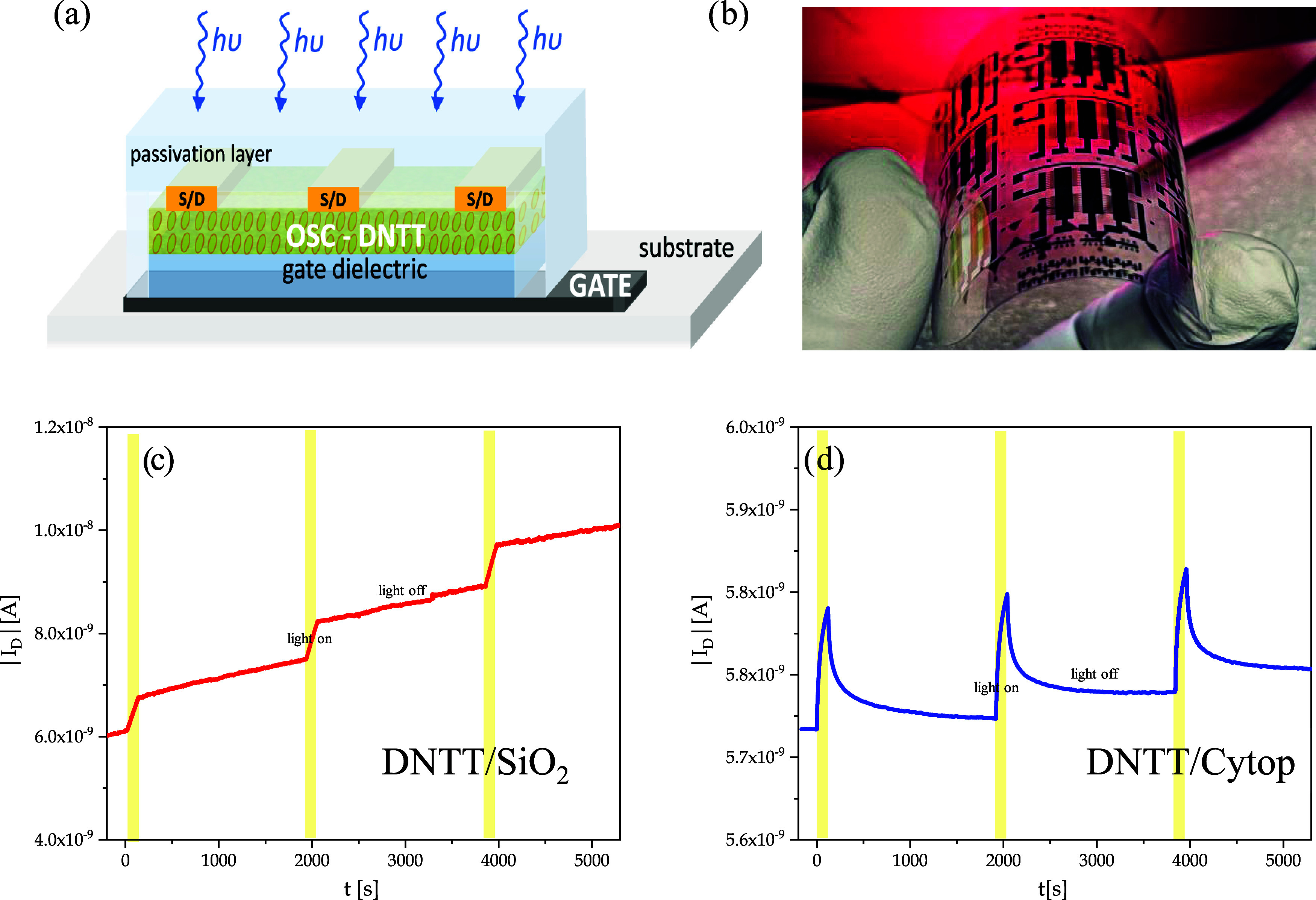
Characterization of organic phototransistor (OPT) devices. (a) Phototransistor scheme (out of scale); (b) DNTT/Cytop OPT flexible sample picture; (c, d) Dynamic multiple photoresponse of the DNTT/SiO_2_ and DNTT/Cytop OPT devices, respectively, under different irradiations at λ = 450 nm. Exposition time, *t*_exp_ = 2 min. Incident power density *P*_opt_ = 2.6 nW/cm^2^.

## 3 Experimental and Methods

### 3.1 Device Fabrication

Two distinct types of OTFTs have been fabricated with staggered Bottom Gate Top Contact (BGTC) configuration at low temperatures (<100 °C) compatible with flexible electronics. For both, the fabrication starts from a commercial substrate of heavily doped Si, which acts as a gate electrode (G), with 100 nm thermal oxide. While in the first OTFT structure, the semiconductor film is grown directly above SiO_2_, acting as a gate dielectric; in the second, the gate dielectric consists of a film of Cytop (600 nm thick) deposited on SiO_2_ by spin-coating technique and annealed at 100 °C for 10 min on a hot plate.^8,62^ The p-type semiconductor is dinaphtho [2,3-b:2,3′-f] thieno [3,2-*b*] thiophene (DNTT, assay 99%, as purchased, Merck), thermally evaporated in high vacuum, at a deposition rate of 0.01 nm/s. The source (S) and drain (D) contacts were made as the last step of the manufacturing process by evaporating in high vacuum a 50 nm film of Au (rate 1 nm/s) through a metal shadow mask, which defines the layout of the devices, with channel lengths (*L*) of 50, 100, 200, and 500 μm and channel width (*W*) of 1000 μm. A 3D sketch of the two different types of OTFT devices is shown in Figure 1b. To compare the electrical performances of the two different device structures, the evaporation of the organic semiconductor layer as well as the metal contacts has been made at the same time to avoid any discrepancy in the process, despite it being very reproducible in all its aspects.

### 3.2 Structural Characterization of DNTT Films

The film morphology was characterized by atomic force microscopy (AFM) measurements carried out in noncontact mode by a psia XE100 system. The crystalline structure of the films was determined by combining GIWAXS and high-resolution X-ray diffraction (XRD) techniques. 2D-GIWAXS diffraction patterns were collected using the 2D Pilatus detector at the XRD1 beamline of the ELETTRA synchrotron facility in Trieste (Italy). The X-ray beam was characterized by an energy of 12.5 keV (corresponding to λ = 1 Å) and a beam size of 200 × 200 μm^2^. The grazing incident angle was fixed at α_i_ = 0.18° to maximize the diffraction signal coming from the semiconducting layer at the top of the substrate. XRD measurements were carried out with a SmartLab Rigaku diffractometer equipped with a rotating copper anode (λ_Cu_ = 1.54184 Å), followed by a parabolic mirror to collimate the incident parallel beam and a series of variable slits placed before and after the sample to control the beam size and detector acceptance, respectively. The beam resolution was 0.01° and 0.1° for the out-of-plane and in-plane measurements, respectively.

### 3.3 Electrical Measurements of OTFTs

The pristine devices were electrically characterized at room temperature using a variable temperature microprobe system, provided by MMR Technologies Inc., connected to Keithley 236 and Keithley 2635 source/meter units. To avoid electrical characteristic variations induced by the absorbed light, the white light of the probe station was filtered by a Schott Glass Technologies KV 600 filter, which cuts the wavelength below λ = 600 nm, preventing optical absorption in the DNTT layer.^42^ Once the devices have been contacted, they are kept in dark for at least 24 h before performing the electrical measurements, holding this condition for the whole time of the electrical characterization. These procedures are used to completely exclude light effects in the reported measurements, since they can deeply influence the electrical characteristics as already demonstrated in the applications of similar devices for organic photo transistors (OPTs).^42^ Low-frequency noise measurements were carried out by the high-sensitivity noise measurement system reported in ref 63 and schematized in Figure 14.

**Figure 14:**
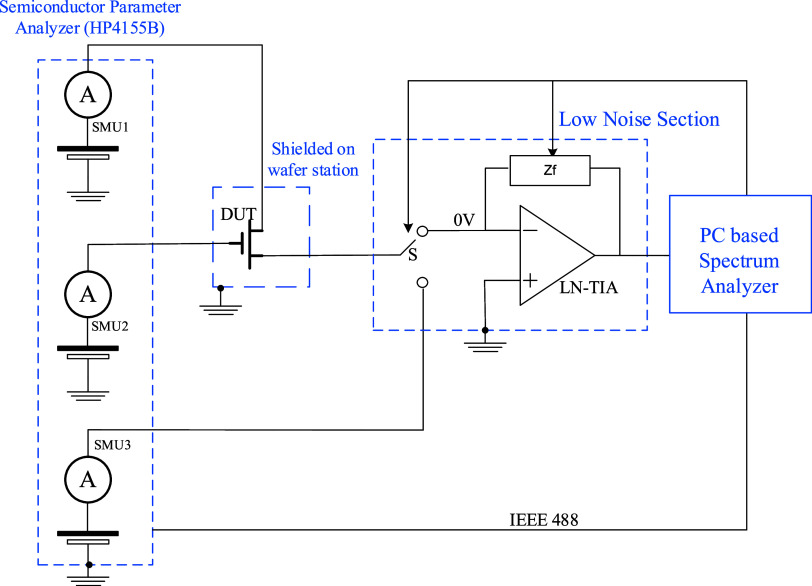
Simplified schematic of the measurement system for low-frequency noise measurements. The bias is applied to the device under test (DUT) through channels SMU1 and SMU2 of the semiconductor parameter analyzer HP4155B. Current channel fluctuations are amplified by a high-sensitivity low-noise transimpedance amplifier (LN-TIA) and measured by a PC-based spectrum analyzer. The DC current is monitored prior to each noise measurement by a third SMU channel (switch S connected to SMU3) to check for device degradation and to set the proper LN-TIA gain (Zf) for subsequent noise measurement.

During noise measurements, the device under test (DUT) is biased in linear regime (*V*_DS_ = −1 V) using two SMU channels, for gate and drain, respectively, of the semiconductor parameter analyzer HP4155B, while the fluctuations of the channel current *I*_*D*_ are measured by a high-sensitivity low-noise transimpedance amplifier (LN-TIA) connected at the source. Prior to each noise measurement, we perform a DC current measurement to check for any device degradation and select the appropriate gain (Zf) for the transimpedance stage. Noise measurements start after an initial hold time of 30 s, necessary to minimize the impact of any displacement currents. Voltage fluctuations at the output of LN-TIA are sampled by a PC-based spectrum analyzer at a sampling frequency of 2 kHz and the power spectral density (PSD) is calculated by averaging PSD of 10 time windows, each containing 32K points. Noise measurements, for each device, are repeated at different gate voltages, from 0 to −30 V.

### 3.4 Density Functional Theory Calculations

All DFT calculations related to DNTT/SiO_2_ were conducted using the Quantum ESPRESSO simulation package.^64^ We employed the Perdew–Burke–Ernzerhof (PBE) functional within the generalized gradient approximation (GGA)^65^ to address the electronic exchange-correlation effects. van der Waals (vdW) interactions, crucial for the adsorption process due to their significant contribution to long-range electron correlation, were taken into account utilizing DFT-D3.^66^ The projector augmented wave (PAW) method^67^ was used to represent ionic core–valence electron interactions. The energy cutoff for the plane-wave basis was set to 450 eV. Atomic positions were relaxed until the magnitude of the Hellmann–Feynman forces on each moving atom was less than 0.02 eV/Å. In the electronic calculation, sampling of the Brillouin zone was restricted to the Γ-point only.

In this study, we employed a hydroxylated amorphous silica (a-SiO_2_) model derived from a previous work.^68^ Our investigation focused on the adsorption properties of DNTT molecules on the silica surface having a silanol density of 5.34 OH/nm^2^, with various adsorption sites, including vicinal and isolated silanols. We first optimized the bare surface before examining the adsorption behavior of DNTT. For the adsorption setup, DNTT molecules were positioned in horizontal and vertical orientations, on the surface. We began with a single molecule, representing minimal coverage, before increasing the number of molecules to 16 to represent full coverage. For the creation of the layer of DNTT molecules, we used the smallest crystalline cell of DNTT corresponding to the herringbone structure. This structure is experimentally demonstrated in this work and has been observed in other studies as well. During the relaxation process, the atoms of the surface below 5 Å were kept fixed, while atoms above this region and the DNTT molecules were allowed to relax. Additional details regarding the SiO_2_ surface and DNTT layers can be found in S4.

The charge density difference was computed as the difference in the electronic densities of the relaxed DNTT/SiO_2_ system and the two separate systems containing the separated SiO_2_ and DNTT at the geometry of the combined system.

The energetics were analyzed using the adsorption energy defined by

3 $$E_{\mathrm{ads}}=\frac{E\left(S{\left[D\right]}_{x}\right)-E\left(S\right)-E\left({\left[D\right]}_{x}\right)}{n}$$

where *E*(S­[D]_*x*_) is the total energy of the full system (surface + adsorbed molecules), *E*(S) is the total energy of the isolated surface, and *E*([D]_*x*_) is the total energy of a single isolated molecule. In cases where more than one molecule is adsorbed, *E*([D]_*x*_) equals the total energy of a single isolated molecule in a vacuum multiplied by the number of molecules *n*. Here, *n* represents the number of molecules in the DNTT layer.

To specifically assess the energy interaction between the DNTT molecules and the surface, excluding structural deformation contributions, we used the following equation:

4 $$E_{\mathrm{int}}=\frac{E\left(S{\left[D\right]}_{x}\right)-E\left(S^{*}\right)-E\left({\left[D^{*}\right]}_{x}\right)}{n}$$

where E­(S*) and *E*([D*]_*x*_) are the total energies of the SiO_2_ surface and the isolated molecules, respectively, with their geometries taken directly from the optimized full system. For a DNTT layer, *E*([D*]_*x*_) is the sum of the energies of each individual molecule extracted from the optimized full system.

To quantify the contribution of dispersion interactions in the adsorption process, we calculated the dispersion energy, *E*_D3_, as

5 $$E_{D3}=\frac{E_{D3}\left(S{\left[D\right]}_{x}\right)-E_{D3}\left(S\right)-E_{D3}\left({\left[D\right]}_{x}\right)}{n}$$

where ***E***_**D3**_(**S**[**D**]_***x***_), ***E***_**D3**_ **(S)**, and ***E***_**D3**_ **(**[**D**]_***x***_ **)** represent the DFT-D3 dispersion energies of the DNTT/SiO_2_ system, the SiO_2_ surface, and the isolated DNTT molecules, respectively. For the DNTT layer, ***E***_**D3**_ **(**[**D**]_***x***_ **)** is the sum of the dispersion energies of each isolated molecule. All energies are calculated for their relaxed geometries. Following an approach from our previous work,^68,69^ we decompose the adsorption energy into different components. Details can be found in section S4.

## 4 Conclusions

In conclusion, we fabricated OTFT devices based on DNTT thin film as a semiconductor material grown on two different substrates, SiO_2_ and the Cytop, respectively, and characterized them by means of electrical and physical/morphological analysis. From the prospective electrical characterization, we measured higher field-effect mobilities for DNTT/Cytop devices and consequently investigated the structure of DNTT films on both substrates to get further insights. From the AFM analysis, in both cases, the DNTT molecules settle and form ordered and layered grains which are laterally larger for DNTT/SiO_2_ films than for DNTT/Cytop ones. XRD measurements evidence that for both films there is a large order of molecules with their longest axis perpendicular to the substrate, as confirmed also by ab initio DFT simulation where we demonstrate that DNTT prefers to stack vertically from the very first layers for the DNTT/SiO_2_ case. Indeed, XRD measurements show that, only for the DNTT/SiO_2_ film, there is also a component of ″horizontal″ molecules with respect to the substrate present in the whole thickness of the semiconductor film, starting from the interface with the dielectric. From numerical simulations on the transfer characteristics, we found that the horizontally ordered component of DNTT molecules in DNTT/SiO_2_ films is compatible with a more defective region found in the connections between DNTT grains, thus explaining the reduced effective mobility for DNTT/SiO_2_ OTFT devices. The higher defectiveness of DNTT/SiO_2_ devices is also confirmed by high-sensitivity, low-frequency noise measurements. Finally, we reported the advantages of using a DNTT/Cytop structure compared with DNTT/SiO_2_ also with regard to the performances of optical detectors.

## Supplementary Material



## Notes

Electrical characteristics at high V_DS_; AFM analysis: other considerations; Deeper analysis of XRD characterization with more graphics; Details on ab initio calculations; Details on TCAD simulations (PDF)
