# Supplementary material for: From Structure to Performance: The Critical Role of DNTT Morphology in Organic TFTs
Source: ACS Appl Mater Interfaces. 2025 Jun 18;17(26):38305–20. doi: 10.1021/acsami.5c07824 (PMC12232279; doi:10.1021/acsami.5c07824)
Supplement: Supplementary file 1 [file am5c07824_si_001.pdf]

Supporting Information

From Structure to Performance: The Critical Role of DNTT Morphology in Organic TFTs

Mattia Scagliotti[1], Antonio Valletta[1], Silvia Milita[2], Luigi Mariucci[1], Gino Giusi[3], Hussam Bouaamlat[4,5], Ari Paavo Seitsonen[6], Paolo Branchini[5], Luca Tortora[4,5], and Matteo Rapisarda[1].*

1 CNR-IMM, Institute for Microelectronics and Microsystems IMM, 00133, Rome, Italy

2 CNR-ISMN, Institute of nanostructured materials ISMN, 40129, Bologna, Italy

3 University of Messina, Engineering department, 98158, Messina, Italy

4 Roma Tre University, Department of Science, 00146, Rome, Italy

5 INFN Roma Tre, Rome, 00146, Italy

6 Department of Chemistry, École Normale Supérieure, 75005, Paris, France

*matteo.rapisarda@cnr.it

S1. Electrical Characterization

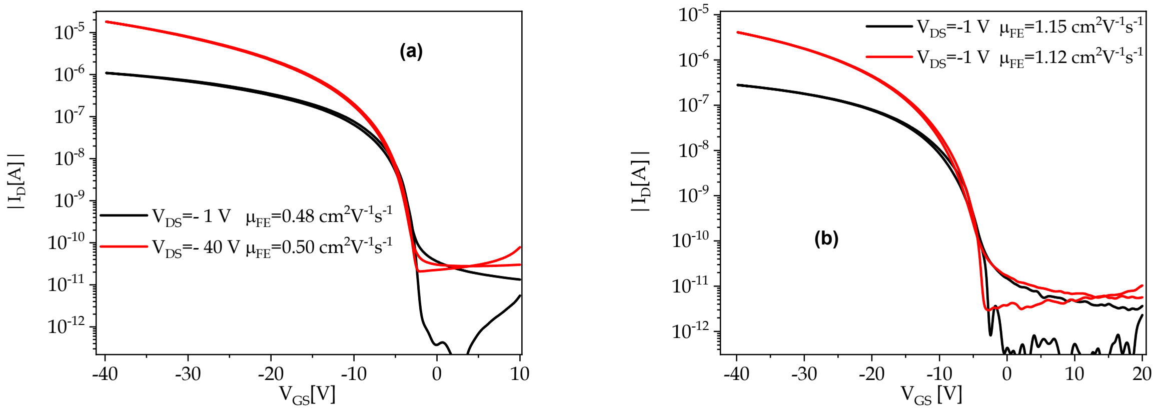

Figure S1: (a) DNTT/SiO_2 device I_D vs V_{GS} characteristics in linear (V_{DS}=-1V) and in saturated (V_{DS}=-40V) regime; **(b)** DNTT/Cytop device I_D vs V_{GS} characteristics in linear (V_{DS}=-1V) and in saturated (V_{DS}=-40V) regime

S2. Atomic Force Microscopy measurements

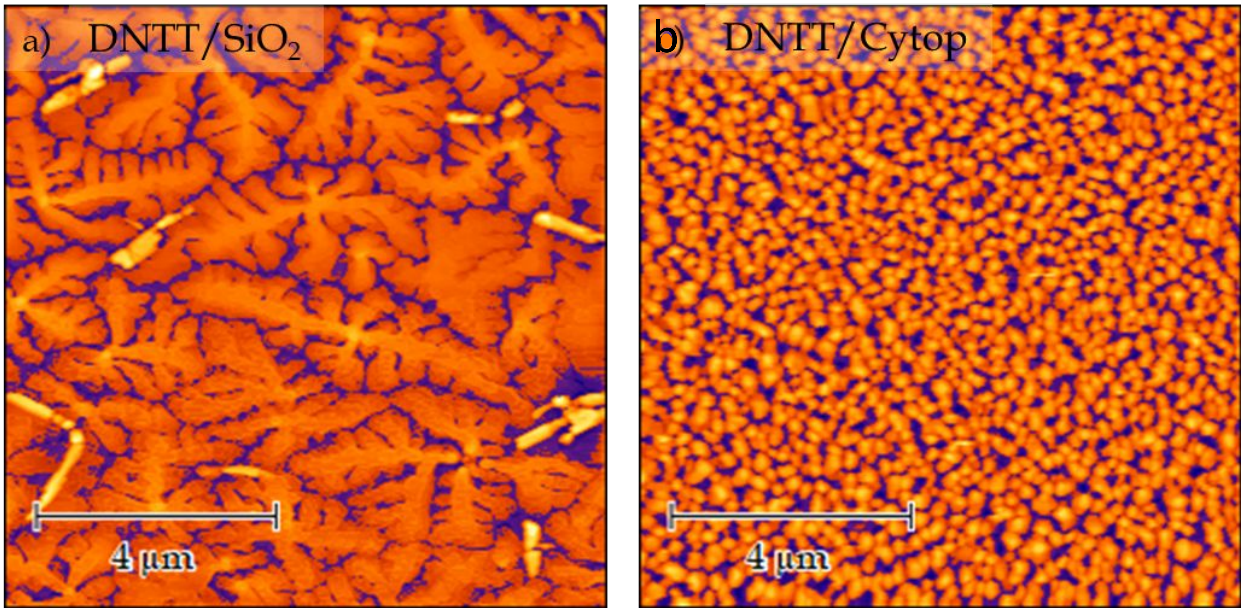

Figure S2: 10 μm x 10 μm AFM topographic images of **a)** DNTT/SiO_2 and **b)** DNTT/Cytop samples with a thickness of 50 nm. The blue area corresponds to a mask applied based on the profile of the grains and their respective heights.

In many cases a dendritic structure does not favor the transport of charge in the plane parallel to the substrate since the grains in this case have a very large perimeter/area ratio unlike a more regular structure which has much fewer grain edges for the same area. The blue mask in Figure S2 was applied via software following the same criterion with the same parameters. It is therefore related to the grain boundaries. The area and volume masks are very similar for both samples. This aspect cannot justify a mobility of lower charges for DNTT/SiO_2 samples compared to DNTT/Cytop samples as shown in Figure 2, although they have much larger lateral grain sizes.

S3. X Ray Diffraction Measurements

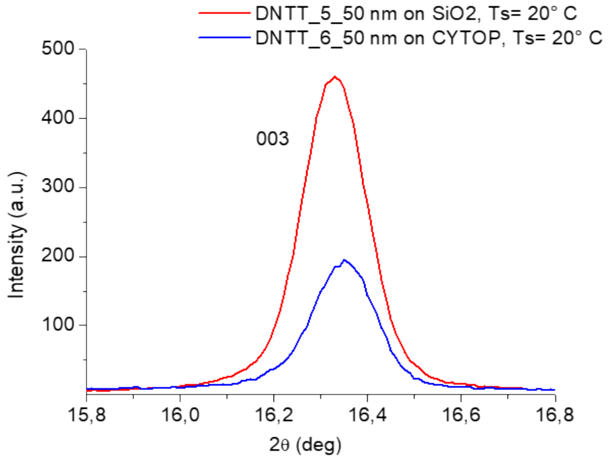

At Ts=20°C

on CYTOP the specular diffracted intensity is lower than on SiO_2 but the films have the same crystalline phase (d-spacing) and vertical coherence length (FWHM):

DNTT/SiO_2: 2θ=16.33°, FWHM= 0.14°

DNTT/CYTOP: 2θ=16.35°, FWHM= 0.13°

Figure S3: HR specular measurements *on (003) peak, on DNTT films of 50 nm grown on SiO_2 and on Cytop at 20°C.*

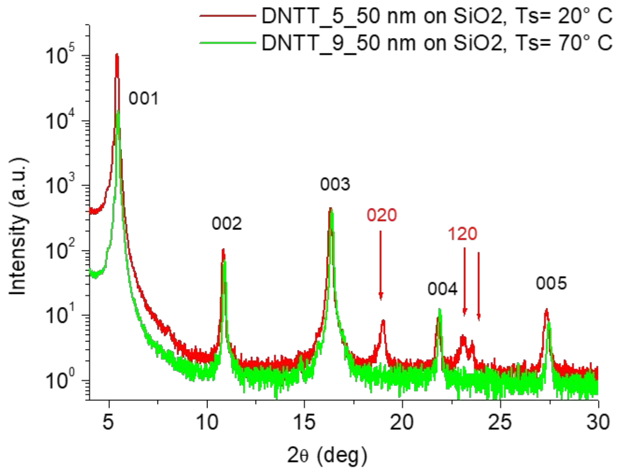

At Ts=70°C the diffraction from crystallites having horizontal orientation disappear.

Figure S4: HR specular measurements *on DNTT films of 50 nm grown on SiO_2 at 20°C and at 70°C.*

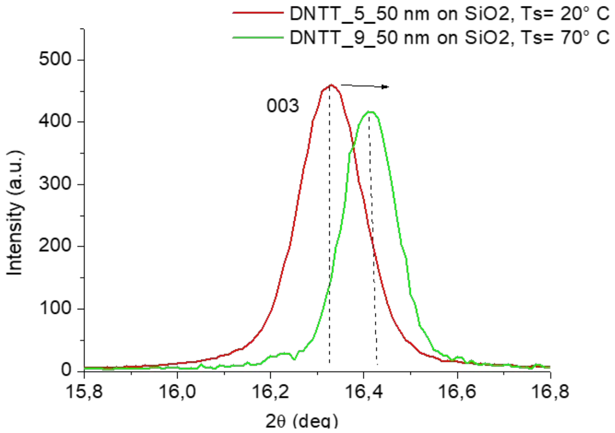

Similar vertical coherence length but different crystalline phase:

Ts=20 °C: 2θ =16.33 deg, FWHM= 0.14 deg (thin film phase)

Figure S5: HR specular measurements *on DNTT films of 50 nm grown on SiO_2 at 20°C and at 70°C.*

Ts=70 °C: 2θ = 16.41 deg, FWHM= 0.11 deg (bulk phase)

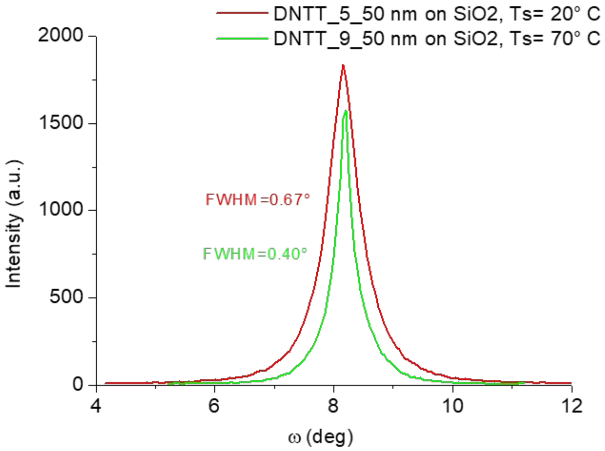

At Ts=20˚C larger mosaicity (0.34 deg) than at Ts= 70˚C (0.2 deg)

Figure S6: *(003) rocking curves on DNTT films of 50 nm grown on SiO_2 at 20°C and at 70°C.*

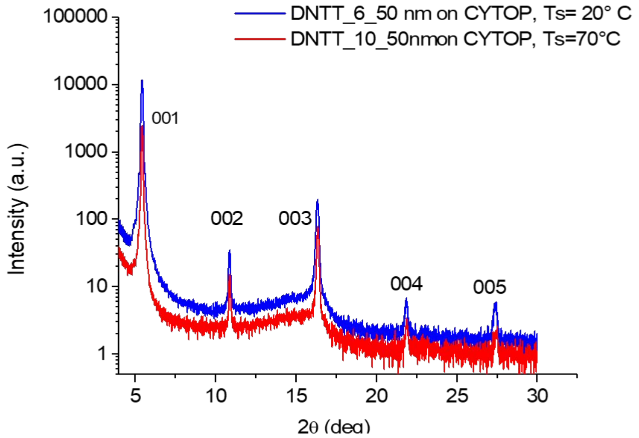

No diffraction from crystallites having horizontal orientation.

***Figure S7: HR specular measurements** on DNTT films of 50 nm grown on Cytop at 20°C and at 70°C.*

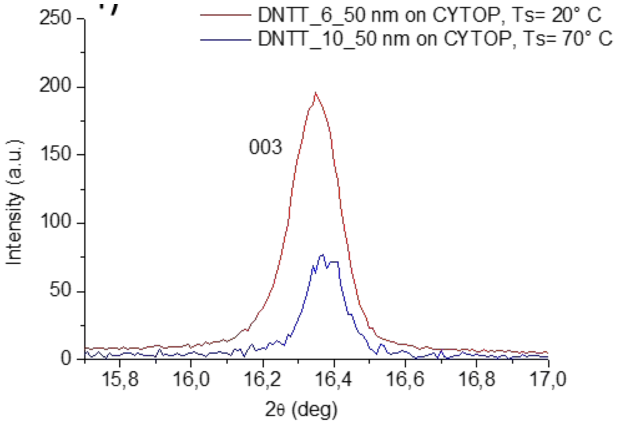

Similar vertical coherence length BUT different crystalline phase:

Ts=20 °C: 2θ =16.35 deg, FWHM= 0.13 deg (thin film phase)

Ts=70 °C: 2θ = 16.38 deg, FWHM= 0.11 deg (bulk phase)

***Figure S8: HR specular measurements** on DNTT films of 50 nm grown on Cytop at 20°C and at 70°C.*

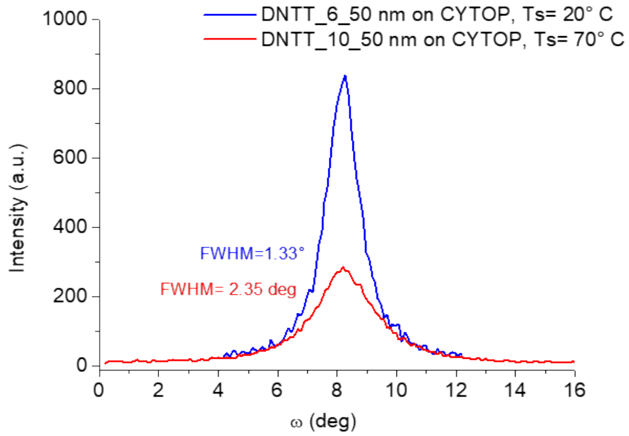

At Ts=20°C mosaicity (0.67 deg) smaller than at Ts= 70°C (1.18 deg)

Figure S9: *(003) rocking curves on DNTT films of 50 nm grown on Cytop at 20°C and at 70°C.*

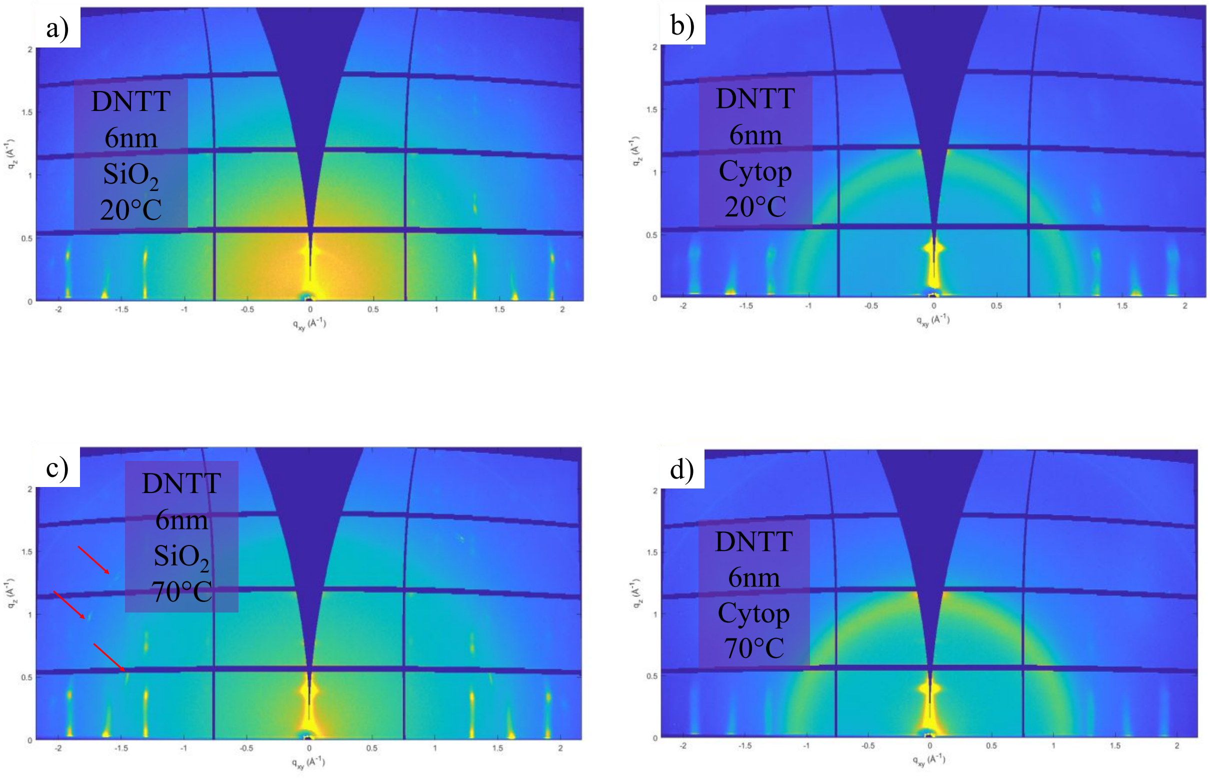

Figure S10: *2D-GIWAXS images of DNTT films:* ***a)*** *DNTT/SiO2 6 nm deposited at 20° C;* ***b)*** *DNTT/Cytop 6 nm deposited at 20° C;* ***c)*** *DNTT/SiO2 6 nm deposited at 70° C;* ***d)*** *DNTT/Cytop 6 nm deposited at 70° C.*

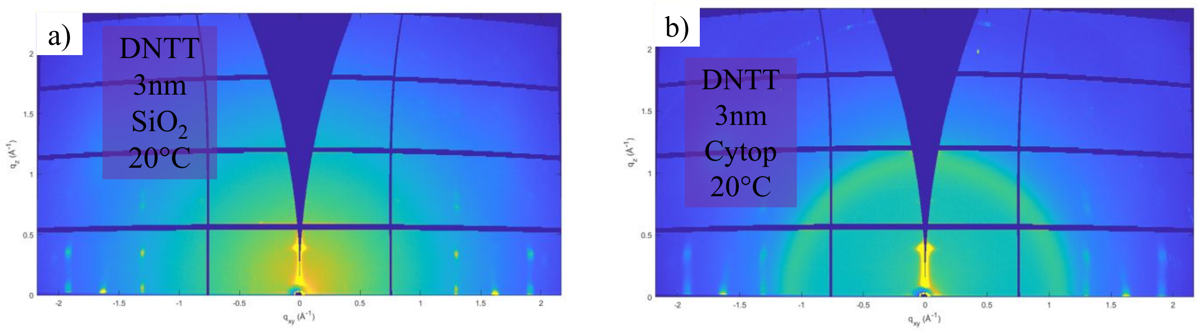

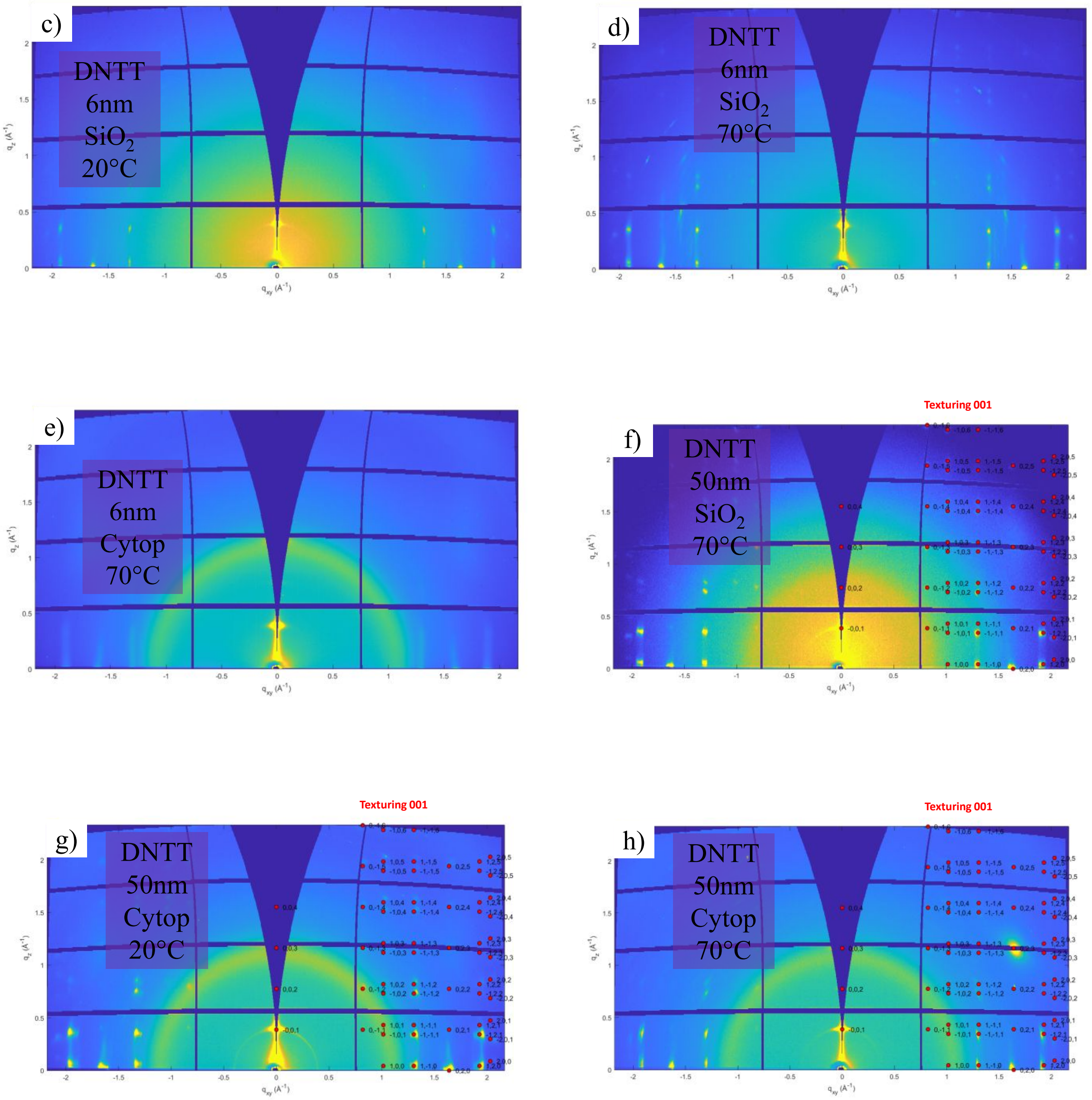

***Figure S11**: 2D-GIWAXS images of DNTT films recorded at 0.05°: **a)** DNTT/SiO2 3 nm deposited at 20° C; **b)** DNTT/Cytop 3 nm deposited at 20° C; **c)** DNTT/SiO2 6 nm deposited at 20° C; **d)** DNTT/SiO2 6 nm deposited at 70° C; **e)** DNTT/Cytop 6 nm deposited at 70° C; **f)** DNTT/SiO2 50 nm deposited at 70° C; **g)** DNTT/Cytop 50 nm deposited at 20° C; **h)** DNTT/Cytop 50 nm deposited at 70° C.*

S4. Ab Initio Density Functional Theory (DFT) Calculations

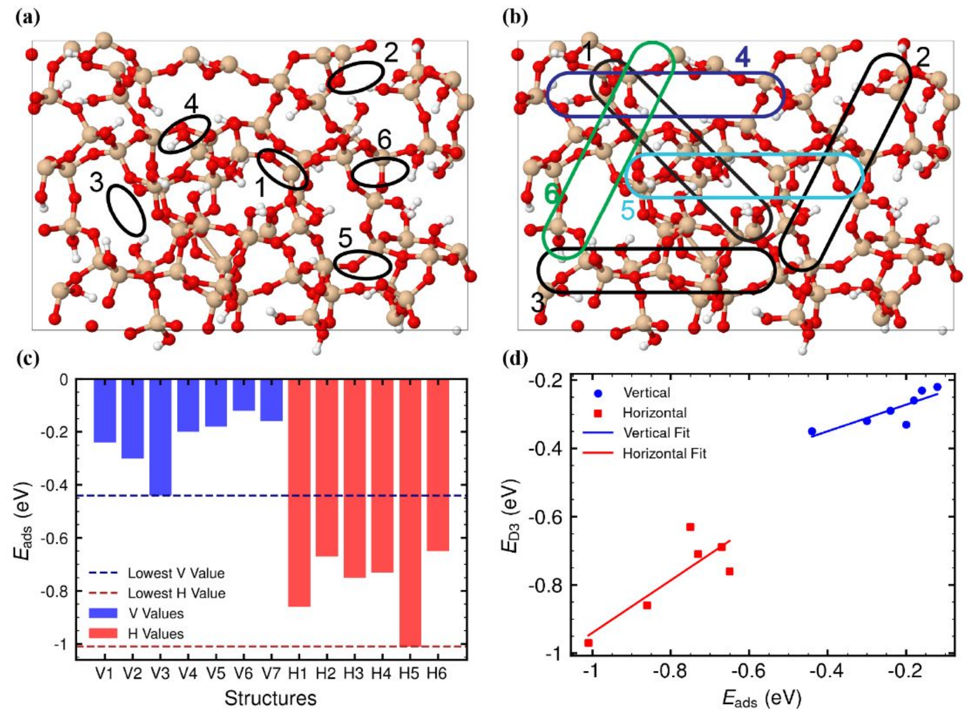

Figure S12: *Different locations of DNTT on top of SiO_2 for vertical* ***(a)*** *and horizontal* ***(b)*** *orientations. The chosen adsorption locations are based on the various adsorption sites available in the SiO_2.* ***(c)*** *Comparison of adsorption energies for different locations of DNTT structures on SiO_2, with vertical orientations (V1–V6) in blue and horizontal orientations (H1–H6) in red.* ***(d)*** *Correlation between adsorption energy and dispersion energy, with blue circles representing vertical orientations and red squares representing horizontal orientations.*

S4.1: The Hydroxylated Amorphous SiO_2 Surface

In this work, we used a model of the amorphous SiO_2 (a-SiO_2) surface from,[1] where they constructed four surface models. Previous work[2] demonstrated that a slab thickness of 12 Å is sufficient to represent an amorphous SiO_2 surface. Based on this, we selected the model with a

thickness of approximately 11 Å and a low roughness of 0.71 Å. Because of the absence of long-range order, we slightly adjusted the b lattice parameter to accommodate the full DNTT layer. The final surface dimensions are 23.74 × 15.76 Å, with a vacuum region of 31 Å in the z-direction. Initially, the surface was relaxed without atomic constraints, allowing the atoms to naturally adopt their bond lengths and angles. The obtained structural properties agree with experimental and computational studies,[3,4] with an average Si–O bond length of 1.643 Å and an average Si–O–Si bond angle of 146.81° (experimental values: 1.61–1.62 Å and 125–165°). The hydroxyl group density was 5.34 OH/nm².

S4.2: DNTT Layer

In the experimental section of this work, IP-GIXD shows diffraction peaks corresponding to the (020), (120), and (110) planes. GIWAXS patterns also indicate that for thinner films (3 nm), DNTT molecules are predominantly oriented vertically, while diffraction signals corresponding to the (0k0) and (hk0) orientations emerge as the film thickness increases. To construct the model of the full coverage to be adsorbed on the amorphous SiO_2 surface, we have created a layer of DNTT molecules. For the creation of the layer, we used the lowest crystalline cell of DNTT, known as the herringbone structure. This structure is experimentally demonstrated in this work and has been observed in other studies as well. Based on these experimental observations, we identify the relevant horizontal facet for adsorption by evaluating the surface energy. The (001) facet is used for adsorption calculations in the vertical orientation, and instead of systematically evaluating all possible horizontal facets of DNTT, we focus only on the most relevant one, the facet with the lowest calculated surface energy. The slabs were generated by cleaving the corresponding facets from the bulk DNTT crystal structure. The surface energy (γ) is calculated using the expression:

$$\gamma = \frac{E_{\text{slab}}\,(N) - NE_{\text{bulk}}}{2A_s} \qquad (1)$$

where $\boldsymbol{E}_{\text{slab}}\,(\boldsymbol{N})$ represents the total energy of an N-layer slab, $\boldsymbol{E}_{\text{bulk}}$ is the bulk total energy (determined as the fitting slope of the $\boldsymbol{E}_{\text{slab}}\,(\boldsymbol{N})$ values to improve accuracy), $\boldsymbol{A_s}$ is the surface area. The factor 1/2 accounts for the two surfaces of the slab. The calculations were performed for N = 3 to 7 layers, with two molecules per layer, using single-point energy calculations only.

The facets exhibiting the lowest surface energy are expected to provide the most stable adsorption configurations. The results in Table S1 indicate that the (001) facet has the lowest energy, followed by the (020). The (020) facet was selected to investigate the adsorption behavior in the horizontal orientation. Two layers were then created from the (001) and (020) facets with dimensions: for (001), a = 24.74 Å and b = 15.32 Å, for (020), a = 16.21 Å and b = 24.74 Å.

Table S1: Surface energies of different facets of DNTT crystal

**Facet**	**Surface energy meV/Å^2**
001	5.23
020	5.32
120	5.63
110	6.09

S4.3: Decomposition of the Energies

In order to deepen our understanding of the physical quantities governing the adsorption process, we derived the decomposition of the adsorption energy into several components. This allows us to determine whether the orientation change is driven primarily by direct molecule–surface interactions or by structural rearrangements of the substrate and adsorbates. We define the adsorption energy as:

$$E_{\mathrm{ads}} = E(\mathrm{S}[\mathrm{D}]_x) - E(\mathrm{S}) - E([\mathrm{D}]_x) \quad (2)$$

where $E(\mathrm{S}[\mathrm{D}]_x)$ is the total energy of the full system (surface + adsorbed molecules), $E(\mathrm{S})$ is the total energy of the isolated surface, and $E([\mathrm{D}]_x)$, is the total energy of a single isolated molecule. In cases where more than one molecule is adsorbed, $E([\mathrm{D}]_x)$ equals the total energy of a single isolated molecule in vacuum multiplied by the number of molecules n. Here, n represents the number of molecules in the DNTT layer.

We define the contributions resulting from the interaction between the adsorbed molecules and the surface, without including the relaxation effects as:

$$E_{\mathrm{int}} = E(\mathrm{S}[\mathrm{D}]_x) - E(S^*) - E([D^*]_x) \quad (3)$$

where $E(S^*)$ is the total energy of the surface within the optimized full system without DNTT layer and $E([D^*]_x)$ represents the energy of the interacting molecules extracted from the optimized full system, calculated as the sum of the energy of each molecule while preserving its geometry from the full system.

We incorporate the interaction energy into the adsorption energy as follows:

$$E_{\mathrm{ads}} = [E(\mathrm{S}[\mathrm{D}]_x) - E(S^*) - E([D^*]_x)] + [E(S^*) - E(\mathrm{S})] + [E([D^*]_x) - E([\mathrm{D}]_x)] \quad (4)$$

and we obtain the decomposition:

$$\boldsymbol{E}_{\mathbf{ads}} = \boldsymbol{E}_{\mathbf{int}} + \boldsymbol{E}_{\mathbf{def}}(\mathbf{S}) + \boldsymbol{E}_{\mathbf{def}}(\boldsymbol{D}) \tag{5}$$

With

$$\boldsymbol{E}_{\mathbf{def}}(\mathbf{S}) = [\boldsymbol{E}(\boldsymbol{S}^{*}) - \boldsymbol{E}(\mathbf{S})] \text{ and } \boldsymbol{E}_{\mathbf{def}}(\boldsymbol{D}) = [\boldsymbol{E}([\boldsymbol{D}^{*}]_{\boldsymbol{x}}) - \boldsymbol{E}([\mathbf{D}]_{\boldsymbol{x}})] \tag{6}$$

Here, $E_{\mathrm{def}}(\mathrm{S})$ represents the energy associated with changes in the surface geometry upon adsorption, and $E_{\mathrm{def}}(D)$ represents the energy cost of altering the molecular geometry.

The molecular deformation energy, $E_{\mathrm{def}}(D)$, can be further decomposed by introducing $E([D^{*}]_{Tx})$ $E([D^{*}]_{Tx})$, the energy of the interacting molecules in their crystalline configuration (the energy of the DNTT layer taken as a whole from the optimized full system). By adding and subtracting $E([D^{*}]_{Tx})$, we obtain:

$$E_{\mathrm{def}}(D) = [E([D^{*}]_{x}) - E([D^{*}]_{Tx})] + [E([D^{*}]_{Tx}) - E([\mathrm{D}]_{x})] \tag{7}$$

where we define:

$$E_{\mathrm{bind}}(D) = [E([D^{*}]_{Tx}) - E([D^{*}]_{x})] \text{ and } \mathrm{E}_{\mathrm{form}} = [E([D^{*}]_{Tx}) - E([\mathrm{D}]_{x})] \tag{8}$$

Thus, the deformation energy becomes:

$$\boldsymbol{E}_{\mathbf{def}}(\boldsymbol{D}) = -\boldsymbol{E}_{\mathrm{bind}}(\boldsymbol{D}) + \boldsymbol{E}_{\mathrm{form}}(\boldsymbol{D}) \tag{9}$$

The binding energy term, $\boldsymbol{E}_{\text{bind}}$ $(\boldsymbol{D})$, describes the intermolecular interactions in the final adsorbed state, while $\boldsymbol{E}_{\text{form}}$ $(\boldsymbol{D})$ represents the energy required to assemble molecules from the gas phase into their crystalline configuration, excluding surface effects.

S4.4: Single molecule

Table S2 presents the decomposition of adsorption and deformation energies into several components. As discussed in the main text, a more negative $\boldsymbol{E}_{\text{ads}}$ indicates higher stability. The horizontal (H5) orientation (−1.01 eV) is more stable than the vertical (V3) case (−0.44 eV), suggesting better surface interaction. $\boldsymbol{E}_{\text{int}}$, which excludes relaxation effects, also favors H5 structure, highlighting its greater stability through the larger contact. In the single molecule cases, the surface undergoes minimal deformation, with $\boldsymbol{E}_{\text{def}}$ $(\boldsymbol{S})$ values of 0.03 eV in V3 and 0.10 in H5. However, $\boldsymbol{E}_{\text{def}}$ $(\boldsymbol{D})$ in the H5 (0.20 eV) is higher than in the V3 structure (0.08 eV). Although the horizontally oriented molecule undergoes greater distortion, its stronger surface interaction still results in higher overall stability compared to the V3 structure. This indicates a trade-off between stronger interaction and increased molecular reorganization. The positive $\boldsymbol{E}_{\text{form}}(\boldsymbol{D})$ primarily reflects the energy cost required to distort the molecule from its optimized gas-phase geometry to the adsorbed geometry, in the absence of intermolecular interactions.

S4.5: Full coverage and orientation shift

When the coverage increases, the Full_V structure becomes favorable than the Full_H structure, as indicated by the more negative $\boldsymbol{E}_{\text{ads}}$, and stronger $\boldsymbol{E}_{\textbf{int}}$. This can be explained by several factors. First, the Full_H structure produces more surface deformation ($\boldsymbol{E}_{\text{def}}(\boldsymbol{S})$ = 0.21 eV) compared to 0.09 eV in the Full_V. This extra cost partly offsets the gains from surface–molecule interactions

in the horizontal orientation, allowing the substrate to accommodate vertically oriented molecules without major structural modification.

Meanwhile, $\boldsymbol{E}_{\mathrm{def}}(\boldsymbol{D})$ is small for both orientations (slightly higher in the vertical case at 0.09 eV). In the Full_V structure, molecules retain a geometry close to their single-molecule form, and neighboring molecules provide additional stabilization. In the Full_H structure, intermolecular interactions also help stabilize each molecule, reducing the need for significant bending or twisting which explain lower distortion compared to the isolated molecule. The $\boldsymbol{E}_{\mathrm{bind}}\,(\boldsymbol{D})$ term, which describes how molecules bind within the adsorbed layer, is more negative in Full_V (−1.38 eV) than in Full_H (−1.21 eV). These stronger molecule–molecule interactions help drive the system toward a vertical configuration at high coverage. As a result, $\boldsymbol{E}_{\mathrm{form}}(\boldsymbol{D})$, the energy change required for isolated molecules to assemble into the adsorbed, interacting state, is also more negative in the vertical configuration (−1.29 eV) than in the horizontal one (−1.17 eV).

Overall, as coverage increases, horizontal orientation imposes a greater penalty through substrate deformation, while vertical orientation is favored due to lower surface deformation, improved formation energy, and stronger molecule–molecule interactions.

***Table S2**: Energetic Characteristics of DNTT and a Layer of DNTT Adsorbed on SiO_2. Adsorption energy (E_{ads}), Interaction energy (E_{int}), Dispersion energy (E_{D3}), the molecule-molecule interaction (E_{bind}), Deformation energy of the surface $E_{def}(S)$, Deformation energy of the molecules $E_{def}(D)$, and the energy to assemble molecules from the gas phase into their crystalline configuration $E_{form}(D)$. All energies presented in the table are given in eV per molecule.*

Structures	E_{ads}	E_{int}	E_{D3}	E_{bind}(D)	E_{def}(S)	E_{def}(D)	$E_{\text{form}}(D)$
V3	-0.44	-0.55	-0.35	0	0.03	0.08	0.08
H5	-1.01	-1.32	-0.97	0	0.10	0.20	0.20
Full_V	-1.41	-1.51	-1.59	-1.38	0.09	0.09	-1.29
Full_H	-1.34	-1.39	-1.52	-1.21	0.21	0.04	-1.17

S5. TCAD simulation

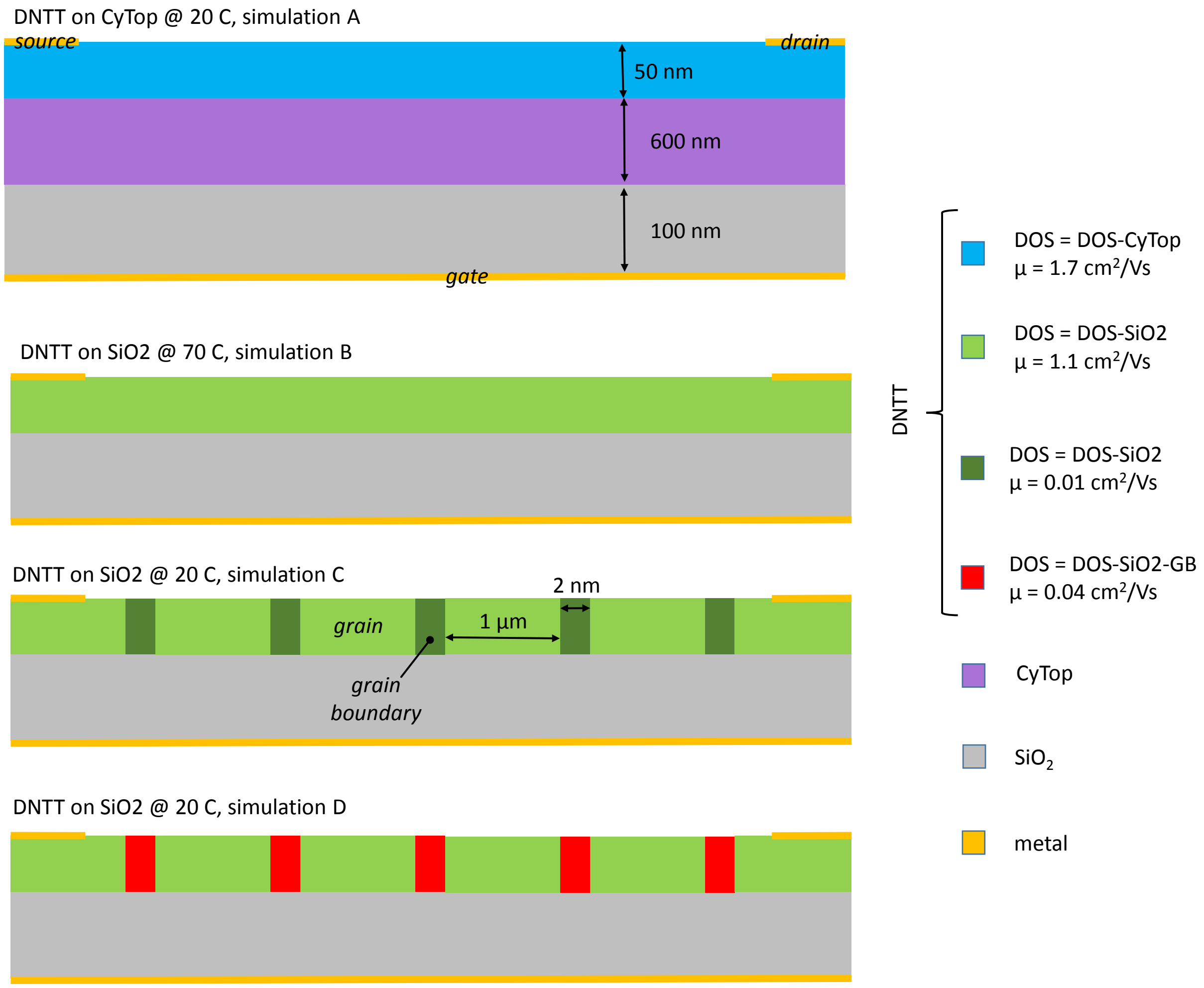

Figure S13: *Schematics (not in scale) of the structures used in the TCAD simulations. Structures for simulations A and B (DNTT grown on CyTop at 20 °C and DNTT grown on* SiO_2 *at 70 °C, respectively) assume a uniform active layer. Structures for simulations C and D, both aiming at the reproduction of the electrical characteristics of OTFTs with DNTT grown on* SiO_2 *at 20 °C, assume a non-uniform active layer with grain boundaries 2 nm wide separating grains 1 μm long, as suggested by the X-Ray diffraction analysis and the DFT results.*

Table S3: *parameters value used in TCAD simulations. The DOS in bandgap are reported in Figure 12d of the main text.*

	**DNTT**	
ε_{DNTT}	DNTT relative dielectric constant	3.0
χ_0	Electron affinity	2.4 eV
E_g	Bandgap	3.0 eV
$N_{c,300}$	Effective density of states in conduction band at T = 300 K	1×10^{21} cm^{-3}
$N_{v,300}$	Effective density of states in valence band at T = 300 K	1×10^{19} cm^{-3}
σ_e	electron cross section for donor states	1×10^{-15} cm^2
σ_h	hole cross section for donor states	1×10^{-17} cm^2
$\mu_{DNTT/CyTop}$	hole band mobility for DNTT grown on SiO_2	1.7 cm^2/Vs
$\mu_{DNTT/SiO2}$	hole band mobility for DNTT grown on SiO_2	1.1 cm^2/Vs
μ_{GB} (sim. C)	hole band mobility for DNTT grain boundaries (used in simulation C)	0.01 cm^2/Vs
μ_{GB} (sim. D)	hole band mobility for DNTT grain boundaries (used in simulation D)	0.04 cm^2/Vs
	**Insulators**	
ε_{CyTop}	CyTop relative dielectric constant	2.25
ε_{SiO2}	SiO_2 relative dielectric constant	3.9
	**Metal (Gold)**	
Φ_M	Metal work function	5.15 eV

References

[1] D. Vörös, A. Angeletti, C. Franchini, S. Mai, and L. González, “Adsorption of 4-(N,N-Dimethylamino)-4′-nitrostilbene on an Amorphous Silica Glass Surface,” *J. Phys. Chem. C*, vol. 127, no. 47, pp. 22964–22974, Nov. 2023, doi: 10.1021/acs.jpcc.3c05552.

[2] C. S. Ewing, S. Bhavsar, G. Veser, J. J. McCarthy, and J. K. Johnson, “Accurate Amorphous Silica Surface Models from First-Principles Thermodynamics of Surface Dehydroxylation,” *Langmuir*, vol. 30, no. 18, pp. 5133–5141, May 2014, doi: 10.1021/la500422p.

[3] R. L. Mozzi and B. E. Warren, “The structure of vitreous silica,” *J Appl Cryst*, vol. 2, no. 4, pp. 164–172, Oct. 1969, doi: 10.1107/S0021889869006868.

[4] S. Perez-Beltran, G. E. Ramírez-Caballero, and P. B. Balbuena, “First-Principles Calculations of Lithiation of a Hydroxylated Surface of Amorphous Silicon Dioxide,” *J. Phys. Chem. C*, vol. 119, no. 29, pp. 16424–16431, Jul. 2015, doi: 10.1021/acs.jpcc.5b02992.
